# Comprehensive dissection of variation and accumulation of free amino acids in tea accessions

**DOI:** 10.1093/hr/uhad263

**Published:** 2023-12-13

**Authors:** Rong Huang, Zhihua Wang, Weiwei Wen, Mingzhe Yao, Haoran Liu, Fang Li, Shuran Zhang, Dejiang Ni, Liang Chen

**Affiliations:** Key Laboratory of Biology, Genetics and Breeding of Special Economic Animals and Plants, Ministry of Agriculture and Rural Affairs; Tea Research Institute of the Chinese Academy of Agricultural Sciences, Hangzhou 310008, China; College of Horticulture and Forestry Science, Huazhong Agricultural University, Wuhan 430070, China; Key Laboratory of Biology, Genetics and Breeding of Special Economic Animals and Plants, Ministry of Agriculture and Rural Affairs; Tea Research Institute of the Chinese Academy of Agricultural Sciences, Hangzhou 310008, China; College of Horticulture and Forestry Science, Huazhong Agricultural University, Wuhan 430070, China; Key Laboratory of Biology, Genetics and Breeding of Special Economic Animals and Plants, Ministry of Agriculture and Rural Affairs; Tea Research Institute of the Chinese Academy of Agricultural Sciences, Hangzhou 310008, China; Key Laboratory of Biology, Genetics and Breeding of Special Economic Animals and Plants, Ministry of Agriculture and Rural Affairs; Tea Research Institute of the Chinese Academy of Agricultural Sciences, Hangzhou 310008, China; Key Laboratory of Biology, Genetics and Breeding of Special Economic Animals and Plants, Ministry of Agriculture and Rural Affairs; Tea Research Institute of the Chinese Academy of Agricultural Sciences, Hangzhou 310008, China; Key Laboratory of Biology, Genetics and Breeding of Special Economic Animals and Plants, Ministry of Agriculture and Rural Affairs; Tea Research Institute of the Chinese Academy of Agricultural Sciences, Hangzhou 310008, China; College of Horticulture and Forestry Science, Huazhong Agricultural University, Wuhan 430070, China; Key Laboratory of Biology, Genetics and Breeding of Special Economic Animals and Plants, Ministry of Agriculture and Rural Affairs; Tea Research Institute of the Chinese Academy of Agricultural Sciences, Hangzhou 310008, China

## Abstract

Free amino acids (FAAs) positively determine the tea quality, notably theanine (Thea), endowing umami taste of tea infusion, which is the profoundly prevalent research in albino tea genetic resources. Therefore, 339 tea accessions were collected to study FAAs level for deciphering its variation and accumulation mechanism. Interestingly, alanine (Ala) and Thea which had the highest diversity index (*H′*) value among three varieties of *Camellia sinensis* (L.) O. Kuntze were significantly higher than wild relatives (*P* < 0.05). The intraspecific arginine (Arg) and glutamine (Gln) contents in *C. sinensis var. assamica* were significantly lower than *sinensis* and *pubilimba* varieties. Moreover, the importance of interdependencies operating across FAAs and chlorophyll levels were highlighted via the cell ultrastructure, metabolomics, and transcriptome analysis. We then determined that the association between phytochrome interacting factor 1 (*CsPIF1*) identified by weighted gene co-expression network analysis (WGCNA) and Thea content. Intriguingly, transient knock-down *CsPIF1* expression increased Thea content in tea plant, and the function verification of *CsPIF1* in *Arabidopsis* also indicated that *CsPIF1* acts as a negative regulator of Thea content by mainly effecting the genes expression related to Thea biosynthesis, transport, and hydrolysis, especially glutamate synthase (*CsGOGAT*), which was validated to be associated with Thea content with a nonsynonymous SNP by Kompetitive Allele-Specific PCR (KASP). We also investigated the interspecific and geographical distribution of this SNP. Taken together, these results help us to understand and clarify the variation and profile of major FAAs in tea germplasms and promote efficient utilization in tea genetic improvement and breeding.

## Introduction

Tea plant (*Camellia sinensis* (L.) O. Kuntze) and its wild relatives, evergreen and woody perennial trees, belong to the family Theaceae genus *Camellia* L. with a long history of cultivation [1, 2]. They have been becoming the most important non-alcoholic beverages following wide consumption worldwide that originated in southwestern China [3]. Besides the great economic value of *C. sinensis var. sinensis*, *C. sinensis var. assamica* (Masters) Kitamura, and *C. sinensis* var. *pubilimba* Chang which are the three varieties of *C. sinensis* (L.) O. Kuntze from morphology, the wild species, for instance, *C. crassicolumna* Chang, *C. tachangensis* F. C. Zhang, and *C. taliensis* (W. W. Smith) Melchior are essentially precious owing to their special physiological activity and health function [3, 4]. For commercial and quality value, the secondary metabolites in tea plants are the vital criteria related to pleasant flavors, nutritional value, and numerous health benefits, encompassing polyphenols, catechins, caffeine, theanine, and terpenes [5–7]. Therefore, the evaluation and utilization of tea accessions are fundamentally important for us contributing to the facilitation of the excavation and breeding of new cultivars.

Among them, the distinctive tea cultivars such as albino [8], purple [9], and other variations possess a unique quality devoting the formation and accumulation of valuable secondary metabolites to their function. A higher concentration of theanine (Thea) content in albino tea cultivars, which exhibit abundant white and yellowing tender leaves, contributes to the umami taste of tea fusion but also prevents people from developing various unhealthy conditions including obesity, inflammation, and aging [10]. Thea is synthesized from glutamate (Glu), which is catalyzed by glutamate dehydrogenase (GDH) and glutamate synthase (GOGAT), and ethylamine (EA) from alanine via alanine decarboxylase (AlaDC) as precursors by theanine synthetase (TS) [11–13]. Moreover, pyridoxine biosynthesis (PDX) and γ-glutamyl-transpeptidase (GGT2) were identified recently to participate in the Thea catabolism. Importantly, arginine (Arg), glutamine (Gln), Glu, and alanine (Ala) as the principal free amino acid components in tea plants devoted to nitrogen recycling, remobilization, and assimilation based on their synthesis function for restoration and improvement of mutual balance [14, 15].

Currently, the most extensive progress found that the block of chloroplast development and the deficiency of chlorophylls biosynthesis following insufficient carbon metabolism and nitrogen consumption led to free amino acids (FAAs) accumulation for restoring this balance, especially Thea content in tea plants [16, 17]. In detail, the inhibition of photosynthetic-related gene expression and the decrease of protein abundance involved in chloroplasts led to FAA accumulation in leaves, with protein degradation [18, 19]. Phytochrome interacting factors (*PIFs*) were destabilized under light conditions, interacting with the photoactivated phytochrome molecules responsible for de-repression of the photomorphogenic program [20, 21]. Liebers *et al.* found that degradation of PIF1/PIF3 as chloroplast development repressors was disrupted with alteration of phytochrome-mediated signaling in the *pap8 Arabidopsis thaliana* albino mutant [22]. Therefore, the expression of genes among the Thea hydrolysis pathway was at the lower level for restoring the balance of carbon and nitrogen mechanism [23]. Considering that, chloroplast development is massively impaired in albino tea plants and heterotrophic metabolism comes to the fore, following the accumulation of FAAs to rescue starvation for carbon metabolism and nitrogen consumption in leaves [24, 25]. Meanwhile, Thea acts as a reservoir for nitrogen, delivered by mainly of identified cationic amino acid transporter (CAT) and amino acid permease (AAP) from root to shoot [26, 27].

**Table 1:** The mean free amino acids profile of tea accessions in two years.

Amino Acid (mg/g)	*C. sinensis var. sinensis* (*n* = 206)	*C. sinensis var. assamica* (*n* = 58)	*C.* *sinensis* var. *pubilimba* (*n* = 25)	Wild relatives (*n* = 29)
Arg	1.70 ± 0.78a	1.21 ± 0.50c	1.57 ± 0.73ab	1.18 ± 0.51bc
Gln	3.29 ± 1.46a	1.80 ± 1.17b	2.93 ± 1.31a	1.29 ± 0.74b
Glu	2.21 ± 0.67a	2.33 ± 0.69ab	2.09 ± 0.55ab	1.88 ± 0.68b
Ala	0.37 ± 0.14a	0.32 ± 0.15b	0.31 ± 0.10b	0.24 ± 0.10c
Thea	13.90 ± 3.63a	12.33 ± 4.80a	14.51 ± 4.41a	10.0 ± 5.03b

The free amino acids content of each accession was the mean of two years. Ala, L-Alanine; Arg, L-Arginine; Gln, L-Glutamine; Glu, L-Glutamate; Thea, L-Theanine. Means within each line that are followed by the different letter are significantly different at *P* < 0.05, based on Tukey’s honestly different significance test.

Furthermore, the rapid progress of metabolite-based transcriptome association technologies has facilitated gene expression profiling involved in different metabolite pathways to mine the crucial function genes. Taken together with the transcriptome, weighted gene co-expression network analysis (WGCNA) is the intensive tool to reflect the expression pattern of functional indentation [28, 29]. Cheng *et al*. identified the transcription factor *CsWRKY40* regulating the hydrolysis of Thea by activating the *CsPDX2.1* promoter with this method [30]. In addition, single nucleotide polymorphisms (SNPs), a prominent molecular marker for assisted breeding, are widely used in improvement of efficiency of tea breeding by evaluating and identifying the desirable alleles in view of functional genes [31]. In this study, to identify the important parameters for underlying accumulation mechanism of FAAs, such as chlorophyll abundance, cell ultrastructure, metabolites, and transcriptions, we screened and clarified the FAA variation of a diversity of 339 tea accessions in where Thea had the highest diversity index (*H′*) value. We further revealed the negative associations between chlorophyll and FAA levels. Importantly, we validated the association of differentially expressed genes (DEGs) based on our previous QTL mapping results for FAA traits [32]. Meanwhile, the crucial gene phytochrome interacting factor 1 (*CsPIF1*) was excavated, combined with WGCNA and identified its negative correlation with the Thea content by response to shade and expression analysis among different cultivars and tissues experiments. Furthermore, transient knock-down expression in tea plants and *CsPIF1*-overexpressing in *Arabidopsis* plants fed with ethylamine analysis revealed that *CsPIF1* acts as the negative regulator by mainly regulating the DEGs involved in the regulation of Thea biosynthesis, transport, and hydrolysis, especially glutamate synthase (*CsGOGAT*) and identified a nonsynonymous SNP by Kompetitive Allele-Specific PCR (KASP) related to Thea content with the interspecific and geographical distribution. Our study provides insights into breeding and the economic value improvement of tea plants by genetic interventions.

## Results

### Overview and variation of free amino acids content among tea accessions

FAAs, notably theanine (Thea) usually represent the crucial parameters reflecting tea infusion quality, prompting us to analyse these traits first. The distribution and variation of FAAs are summarized in Table 1. Overall, the average values of arginine (Arg), glutamine (Gln), glutamate (Glu), alanine (Ala), and Thea in *C. sinensis* were all significantly higher than those in the wild relatives (*P* < 0.05), revealing that the wild relatives possess the characteristics of low FAAs level. Among intraspecific accessions, Arg, Gln, and Ala amounts in *sinensis* were highest, with 1.70 mg/g, 3.29 mg/g, and 0.37 mg/g, respectively, while Glu amount in *assamica* (2.33 mg/g) and Thea amount in *pubilimba* (14.51 mg/g) were at the top. Additionally, the mean content of Arg and Gln in both *sinensis* and *pubilimba* were significantly higher than in the *assamica* (*P* < 0.05) variety. Meanwhile, *sinensis* displayed significant difference with *assamica* (0.32 mg/g) and *pubilimba* (0.31 mg/g) varieties in terms of Ala (*P* < 0.05).

The diversity of the tea accessions from different areas are observed in Table 2. The amount of Thea averaged 13.23 (0.02–26.46) mg/g, although there was a very low level in *C. reticulata* Lindl. (‘DS’) and *C. oleifera* Abel (‘YC’). The contents of Gln and Glu were next, averaging 2.83 (0.20–8.62) mg/g and 2.16 (0.38–4.58) mg/g, respectively. However, the content of Ala (0.06–0.92 mg/g) was lower than Arg content, belonging to the minor component. Moreover, most free amino acid components were in normal distribution, indicating that they belong to quantitative traits that are easily susceptible to ambient environment including light, humidity, temperature, and fertilizer, thereby regulating by multiple genes [33, 34], following our previous research [32]. In addition, the diversity index (*H′*) value of Thea was 2.03, followed by Gln (2.00), Glu (1.96), Ala (1.96), validating the feasibility and diversity of FAAs of tea germplasms from the main tea-producing regions. This study helps us to further develop and utilize the valuable and transnormal tea genetic resources in terms of FAAs.

**Table 2:** Distribution and variation of free amino acids in 339 tea accessions.

Amino acid	Max (mg/g)	Min (mg/g)	Median (mg/g)	Mean + SD (mg/g)	Kurtosis	Skewness	*H′*
Arg	4.48	0.78	1.37	1.57 ± 0.74	1.20	2.24	1.77
Gln	8.62	0.20	2.71	2.83 ± 1.55	0.65	0.72	2.00
Glu	4.58	0.38	2.03	2.16 ± 0.77	1.08	0.83	1.96
Ala	0.92	0.06	0.31	0.34 ± 0.15	1.64	1.09	1.96
Thea	26.46	0.02	13.33	13.23 ± 4.39	1.00	−0.26	2.03

The free amino acids content of each accession was the mean of two years.

**Figure 1:**
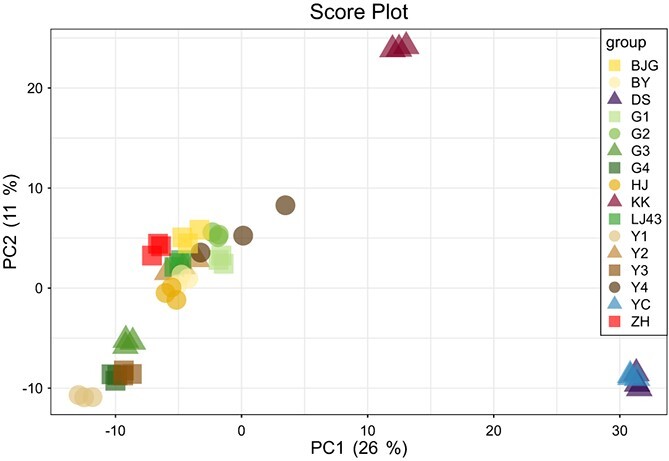
Principal component analysis of metabolites levels among the FAAs difference tea accessions. G1-G4 and Y1-Y4: F1 offspring from amino acids artificial segregating population of *Camellia sinensis* ‘Longjing 43’ (‘LJ43’) × *C. sinensis* ‘Baijiguan’ (‘BJG’); BY: *C. sinensis* ‘Baiye 1’; DS: * C. reticulata* Lindl.; HJ: *C. sinensis* ‘Huangjingya’; KK: *C. sinensis* var. *pubilimba* ‘Kekecha’; YC: *C. oleifera* Abel; ZH: *C. sinensis* ‘Zhonghuang 2’.

Cladistic clustering was performed to dissect the distribution patterns of six components, and the result showed all the samples were divided into three groups: Groups 1, 2, and 3 (Fig. S1, see online supplementary material). Interestingly, *C. sinensis* var. *pubilimba* ‘Kekecha’ (‘KK’), ‘DS’, and ‘YC’, which were at lower level of FAAs, were in the same group (Group 3), illustrating that ‘KK’ may have the potentially related or even similar metabolites with ‘DS’ and ‘YC’ to some degree [35].

### Metabolites profiling for the FAAs difference tea accessions

Given the association mentioned above, we further excavated the various metabolites among the difference of FAAs tea accessions to elucidate their differential metabolic mechanism with three biological duplications, including 22 tea accessions (eight from the amino acids artificial segregating population of ‘Longjing 43’ × ‘Baijiguan’ and their parents, 12 extreme amino acids level from the natural population). For this purpose, we identified, in total 1113 known metabolites by UPLC–MS/MS for all analyses: 87 alkaloids, 101 amino acids and derivatives, 187 flavonoids, 30 lignans and coumarins, 143 lipids, 73 nucleotides and derivatives, 108 organic acids, 214 phenolic acids, 29 tannins, 17 terpenoids, and 119 other metabolites (Table S1, see online supplementary material). The Pearson correlation coefficient of those mix concerned with quality control ranged from 0.99 to 1 (Fig. S2, see online supplementary material), indicating that the data was stable.

A principal component analysis (PCA) was performed, illustrating that PC1 and PC2 explained 26% and 11% of the variation of metabolites, respectively (Fig. 1). ‘DS’ and ‘YC’, which belong to different species, were obviously distributed together to distinguish them from the other tea accessions. Moreover, although ‘KK’ is classified as *C. sinensis* var. *pubilimba*, it was not clustered with other tea plants, revealing it is unique in terms of metabolites, consistent with the results of free amino acids value, promoting us to further analyse from the genetic mechanism. Meanwhile it was not surprising that the offspring of the ‘LJ43’ × ‘BJG’ population were clustered with their parents and three albino cultivars (‘BY’, ‘HJ’, and ‘ZH’).

### Different metabolites correlation and cell ultrastructure difference analysis

**Figure 2:**
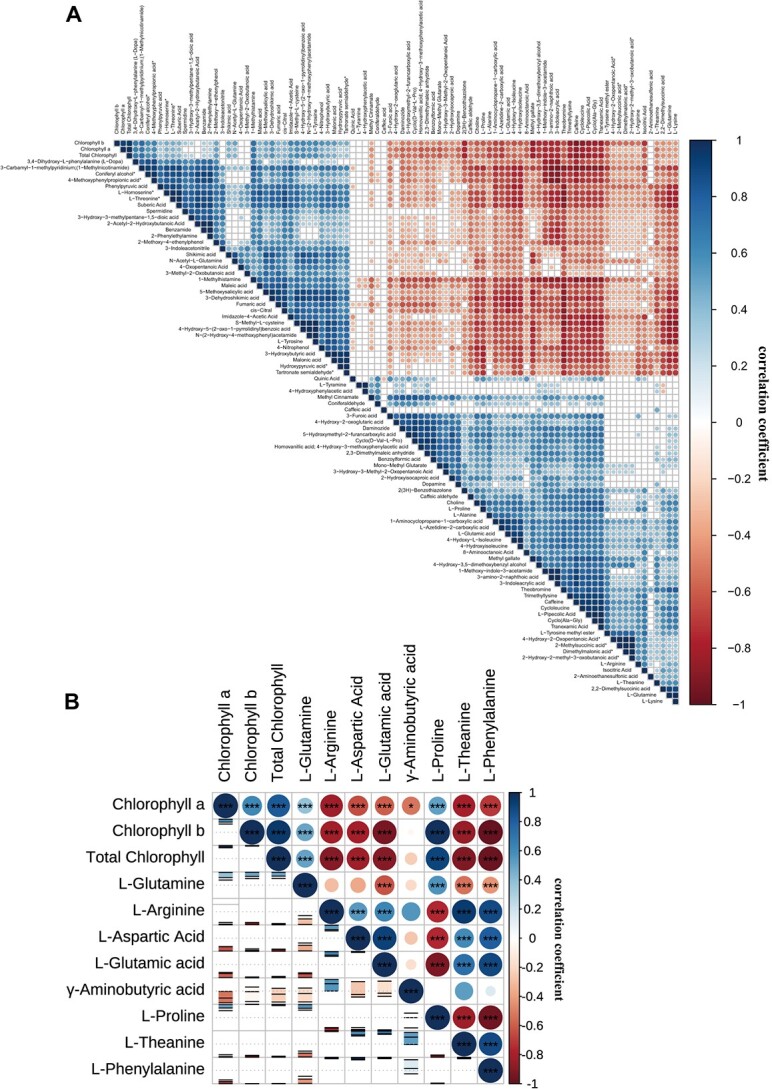
Correlation analysis in the metabolites for FAAs difference tea accessions. **A** Different metabolites correlation analysis. **B** Chlorophyll and free amino acids profiling correlation analysis. Upper displayed correlation coefficient and *P*-value (^*^P ≤ 0.05; ^**^P ≤ 0.01; ^***^P ≤ 0.001). Lower displayed correlation coefficient confidence interval (95%).

To explore the relationship between FAAs and other metabolites, Pearson’s correlation coefficients and associated *P*-values were exhibited for improving metabolic networks. Here, a total of 89 pairs of detected metabolites were highly associated, as their *P*-values were <0.05 (Fig. 2A). Besides, what was worthy of our attention is that the photosynthetic pigments including chlorophyll *a* (Chl a), chlorophyll *b* (Chl b), and total chlorophyll (Chl) showed negative correlation with the substances in the Thea biosynthesis pathway. Of them, Chl a and Chl were significantly and negatively related with Ala (*|r*| > 0.65 and *P* < 0.05), which is the direct precursor of Thea synthesis. The *|r*| and *P*-values of Chl b with Ala and Glu were > 0.55 and *P* < 0.01, respectively. Obviously, Thea which is the quality-related trait in tea plants, has a potential and mutual transformation relationship with chlorophyll.

**Figure 3:**
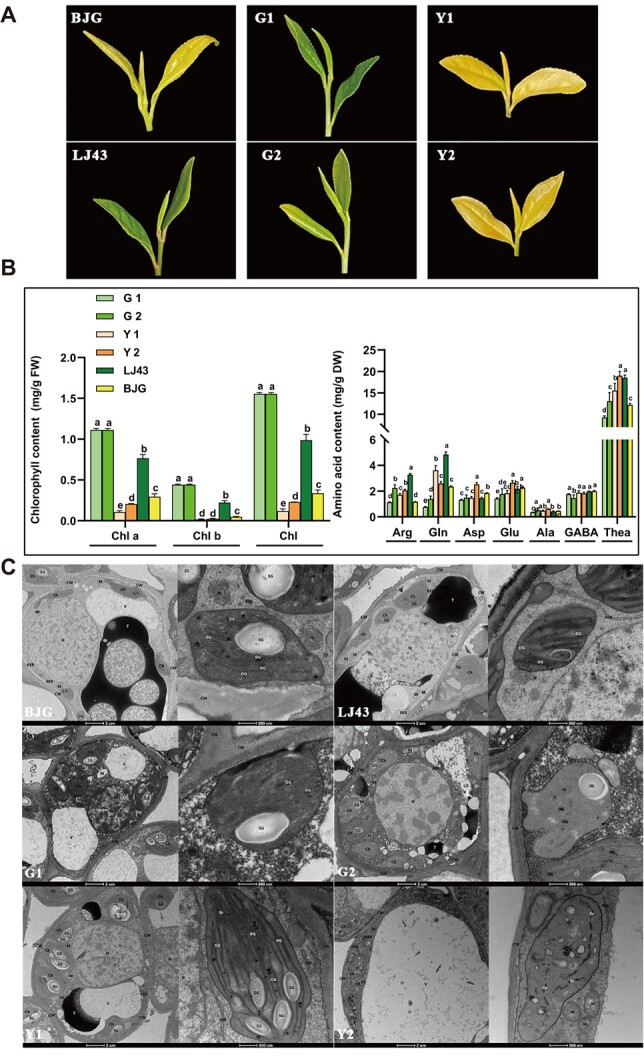
Analysis of cell ultrastructure and metabolites among FAAs segregating offspring and their parents. **A** Phenotypic characteristics of F1 offspring and their parents. **B** Chlorophyll and free amino acids content analysis. **C** Chloroplast ultrastructure analysis. Ch, chloroplast; Gr, granum; OG, osmiophilic granule; SG, starch granule; T, tannin; Th, thylakoid; V, vacuole.

**Figure 4:**
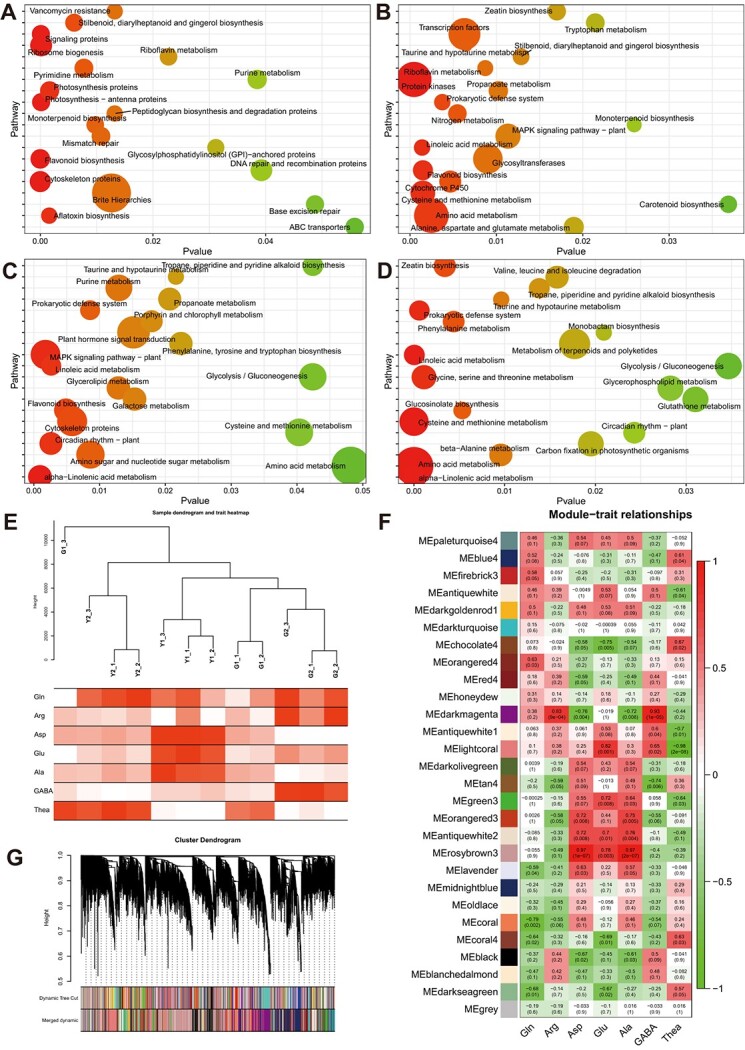
DEGs analysis based on KEGG pathways and weighted gene co-expression network analysis based on the gene expression and the physiological data. **A**–**D** Top 20 enriched KEGG pathways among DEGs in ‘BJG’ vs. G1, ‘BJG’ vs. G2, Y2 vs. G1, and Y2 vs. G2, respectively. **E** Sample dendrogram and trait heatmap based on gene expression and physiological data. **F** Co-expression network analysis in terms of free amino acids content traits. **G** Gene cluster dendrogram with different modules and similar modules merging.

**Figure 5:**
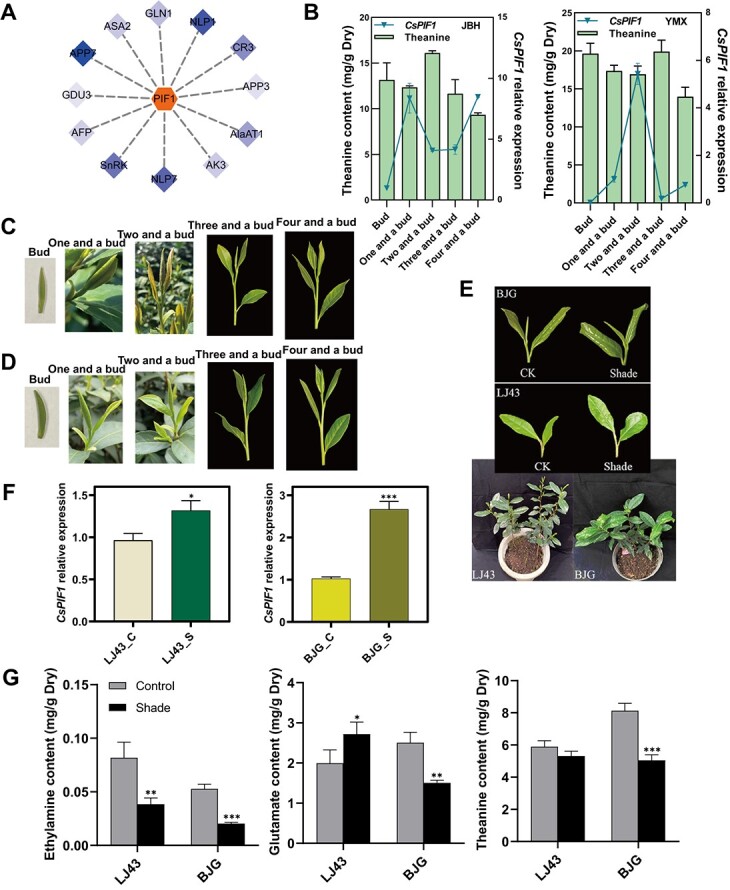
Expression analysis of *CsPIF1* among different tissues and under normal and darkness conditions of tea plants. **A** Weight analysis between *CsPIF1* and genes related to amino acid metabolism. **B** Analysis of *CsPIF1* expression and theanine contents in the different tea cultivars and different tissues. **C** and **D** Phenotypic characteristics of ‘Jianbohuang 13’ and ‘Yuemingxiang’ at different growth stages. **E** Shade treatment in ‘BJG’ and ‘LJ43’. **F** Verification *CsPIF1* expression responds to shade in ‘LJ43’ and ‘BJG’. **G** Free amino acids and ethylamine shift responds to shade in ‘LJ43’ and ‘BJG’. Asterisks above the error bar indicate significant differences (^*^P ≤ 0.05; ^**^P ≤ 0.01; ^***^P ≤ 0.001).

Considering this connection, we further analysed the representative albino cultivars as they possess high Thea and low chlorophyll level characteristics. As expected, there was a significantly negative relationship between chlorophyll and FAA profiles (Fig. 2B). Arg, aspartic acid (Asp), Glu, and Thea were significantly and negatively correlated with Chl b and Chl, with *|r*| > 0.8 and *P* < 0.01. L-Phenylalanine, which shares the precursor substances with Thea, also showed the same association with Chl (*|r*| > 0.9 and *P* < 0.001). In contrast, L-Proline, another branch product of pyruvic acid, presented a strikingly positive correlation with Chl b and Chl (*r* > 0.9 and *P* < 0.05). As such, this result further confirmed our previous results that the levels of chlorophyll and Thea metabolites were opposite trends.

Chlorophyll content usually represents an important parameter reflecting the photosynthetic capacity of tea plants. We therefore further analysed the inner cell structure to uncover the correlation mentioned above. Firstly, the Chl a, Chl b, and Chl contents in albino offsprings (Y1 and Y2) (Fig. 3A) were remarkably lower than that of green ones (G1 and G2) with *P* < 0.01 (Fig. 3B). And contrarily, the levels of Glu, Asp, Gln, and Thea in albino were higher than that of green individuals (Fig. 3B). Therefore, we subsequently observed the cell ultrastructure of these samples using transmission electron microscope (Fig. 3C). The result revealed that G1 and G2 contained abundant chloroplasts (Ch) with tightly stacked grana (Gr), enriched starch granules (SG), ordered osmiophilic granules (OG), and some vacuoles (V). Y1 and Y2 in which Ch transferred albinism with uneven matrix and unobvious grana lamellar structure had fewer volumes of Ch, Gr, and SG. Moreover, there were no obvious changes in capsule structure and the matrix thylakoid was dissolved and disappeared. These observations were in alignment with the shift and association between chlorophyll and FAA content. As such, the results could also be found in their parents ‘LJ43’ and ‘BJG’.

### RNA sequencing, mapping, and DEGs analysis among offspring and their parents

Given the invaluable findings mentioned above, we further dissected the underlying genetic mechanism from transcriptome sequencing analysis. In detail, the clean reads ranged from 32.90 to 63.98 million reads, and the Q20 base percentage was 90.29%, on average after reads evaluation and trimming filters. Subsequently, the clean reads were mapped to the reference genome of *C. sinensis var. sinensis* ‘Shuchazao’ [36]. PCA analysis suggested that the percentages of PC1 and PC2 were 33% and 13%, respectively (Fig. S3, see online supplementary material). Kyoto Encyclopedia of Genes Genomes (KEGG) pathway analysis revealed that the DEGs were most substantially enriched in ‘flavonoid biosynthesis’, ‘photosynthesis proteins’, ‘amino acid metabolism’, and ‘chlorophyll metabolism’ in offspring and their parents mentioned above (Fig. 4A–D). Therefore, we inferred that the results of the enrichment pathway were responsible for both biochemical and physiological phenotypes of distinctive albino cultivar. What is more, we also focused on the DEGs between *Thea* section (‘BY’) with higher level of Thea and non-*Thea* section (‘YC’) where the Thea content can hardly be detected, which were also notably enriched in ‘amino acid metabolism’ (Fig. S4, see online supplementary material).

These findings impelled us to further investigate the functional genes from DEGs among photosynthesis, chlorophyll, and amino acid metabolism. Therefore, we constructed co-expression network analysis (WGCNA) and the visualization of sample dendrogram and trait heatmap was in Fig. 4E to elucidate the relationship between genes expression and physiological traits. The square correlation coefficient (R^2^) value of the parameters log (k) and log [p (k)] were set to be 0.8(≥0.8), and soft-threshold power β = 5 (Fig. S5, see online supplementary material). Subsequently, the gene modules were constructed based on TOM matrices for merging similar modules. Consequently, a total of 28 co-expression modules were identified (Fig. 4G;Fig. S6, see online supplementary material), including 10 modules positively correlated with FAAs (PCC > 0.6, *P* < 0.05), notably ‘rosybrown3’ module with Ala (PCC = 0.97, *P* = 2.0 × 10^−07^), Glu (PCC = 0.78, *P* = 0.003), and Asp (PCC = 0.97, *P* = 1.0 × 10^−07^), and nine modules negatively correlated with FAAs (PCC < −0.6, *P* < 0.05) based on Pearson correlation coefficients (PCC) (Fig. 4F). We then conculcated the gene significance (GS ≥ 0.6) based on module significance (MS) to identify candidate genes (Table S2, see online supplementary material). It is not unexpected that crucial functional genes were not directly involved in Thea metabolism, but mainly regulation of photosynthesis and chloroplast biogenesis. Based on our previous QTL mapping results with the Thea trait [32], we found that the candidate gene phytochrome interacting factor 1 (*CsPIF1*) with transcriptional activation in the dark for inhibiting chlorophyll biosynthesis [37], cationic amino acid transporter 1 (*CsCAT1*), and ATP-dependent zinc metalloprotease FTSH5 (*CsVAR*) involved in the degradation process of D1 protein in photosystem II [38] were identified in the Y2 vs. G1 comparison. Here, the *CsPIF1* was found from rosybrown3 module showing significant correlation with Asp, Ala, and Glu that Pearson correlation coefficients (PCC) were > 0.75 with *P* < 0.01 (Fig. 4F). However, *CsCAT1* was in chocolate 4 modules showing correlation with Thea with PCC < 0.8 and *CsVAR* was found in blanchedalmond module (Table S2, see online supplementary material), which was not related to any amino acid components (*P* > 0.05).

### *CsPIF1* expression analysis and response to shade treatment verification

In order to confirm the relationship between amino acid metabolism and chloroplast biogenesis found above, we identified the *CsPIF1* (PCC > 0.95) showing the highest weight value with *CsAAP7* based on WGCNA analysis (Fig. 5A), which is involved in the uptake of amino acids and contributes to the long-distance transport of Thea [39]. Meanwhile, *CsNLP1* and *CsNLP7*, which modulate the nitrate sensing and metabolism, also had high weight level with *CsPIF1* to reveal the important regulatory role of *CsPIF1* in amino acid metabolism. Therefore, to identify the association, we analysed the *CsPIF1* expression next among different cultivars (‘Jianbohuang 13’ and ‘Yuemingxiang’) (Fig. 5C and D) and different tissues. The result showed that the *CsPIF1* expression levels from bud to ‘four and a bud’ were negatively correlated with Thea accumulation (Fig. 5B).

Furthermore, to ask whether *CsPIF1* responds to shade for effecting the Thea level, we selected the dark-activated *CsPIF1* to detect the expression after shade treatment with the shoots under sunlight condition as the control (Fig. 5E). Finally, the shade treatment significantly increased the expression of *CsPIF1* (Fig. 5F) both in ‘LJ43’ (*P* < 0.05) and ‘BJG’ (*P* < 0.01) with the significant decrease of EA, Glu, and Thea levels in ‘BJG’, and the significant decrease of EA amount in ‘LJ43’ (Fig. 5G). These results suggested that *CsPIF1* may play a negative role in Thea accumulation and can respond to shade for the Thea change that was in agreement with the opposite pattern.

**Figure 6:**
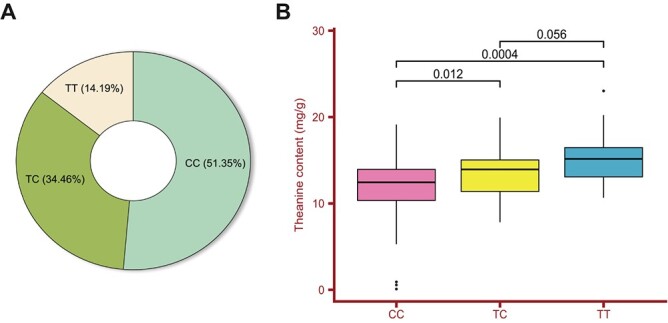
Genotypes of SNP179068992 in 148 tea accessions. **A** The percentages of CC, TC, and TT genotypes. **B** Thea content in tea plants with the three different SNP179068992 genotypes.

### Validation and distribution of the SNPs based on DEGs regulating theanine content

In addition, we further identified the DEGs related to Thea biosynthesis based on results above from a total of 18 SNPs that were tested for reliability by 148 accessions with KASP analysis. Fortunately, a nonsynonymous SNP named SNP179068992 which is located in the coding sequence of glutamate synthase (GOGAT) was found to regulate the Thea content in tea plants [40], with three specific genotypes (CC, TC, and TT) (Table S3, see online supplementary material), and made a substantial contribution to Thea (*P* < 0.01) based on ANOVA (Table S3, see online supplementary material). In detail, the 1016th amino acid is Serine (Ser) or Leucine (Leu) when C or T is located at the SNP179068992 site (Fig. S7, see online supplementary material), respectively, and genotype CC accounted for up to 51.35%, while TT only occupied 14.19% (Fig. 6A), revealing that CC was the dominant genotype at the SNP179068992 site.

Notably, the median Thea values of the tea accessions with the genotype TT (15.16 mg/g) was significantly higher than CC (12.44 mg/g) with *P* < 0.01 (Fig. 6B), and the genotype TC (13.93 mg/g) also had a significant difference with CC (*P* < 0.05). In conclusion, the tea plants with the homozygous genotype TT could accumulate substantially more Thea than those with the genotypes CC and TC. Meanwhile, tea plants in half of the provinces possessed the three different genotypes, including Yunnan, Sichuan, Guangxi, and Fujian, which are rich in tea genetic resources (Table 3), suggesting that it is easier to tap specific resources for high Thea trait in these provinces. Importantly, the SNP179068992 site of offspring G1 and G2 with lower Thea content are CC, and TC in Y1 and Y2 (Fig. 3B; see online supplementary material Table S3), consistent with the correlation between the genotypes and Thea value, further confirming the reliability of SNP179068992 site.

**Table 3:** Geographical distribution of the three genotypes.

Province	Genotypes
Anhui	CQ
Chongqing	CQT
Fujian	CQT
Guizhou	CQ
Guangxi	CQT
Guangdong	CQT
Hubei	CQ
Hunan	CT
Hainan	CT
Jiangxi	CQT
Jiangsu	C
Shanxi	C
Sichuan	CQT
Taiwan	CT
Yunnan	CQT
Zhejiang	CQT

C: CC; Q: TC; T: TT.

### Identifying the relationship between *CsPIF1* and Theanine content

**Figure 7:**
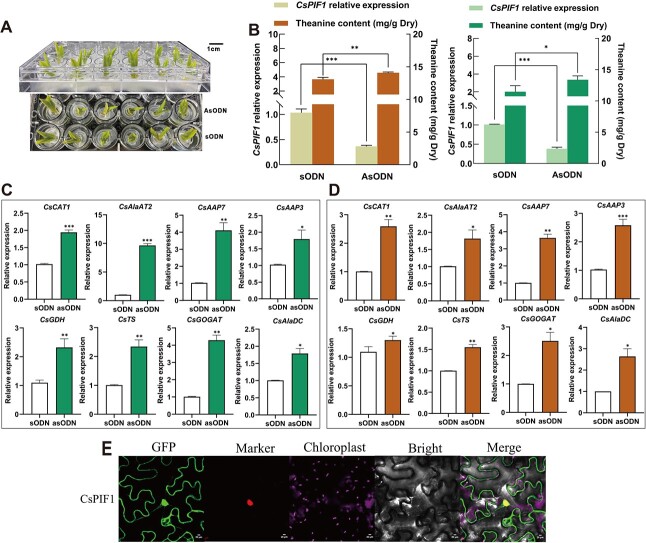
Identification and localization the relationship between *CsPIF1* and Theanine content. **A** and **B** The effect of knockdown of *CsPIF1* expression with asODN on FAAs in ‘BJG’ and ‘LJ43’, oligonucleotide serving as a control. **C** and **D** The analysis of DGEs associated with theanine synthesis, transport, and hydrolysis by transient knockdown *CsPIF1* expression in the new shoots of tea plants. **E***CsPIF1* subcellular localization analysis. Asterisks above the error bar indicate significant differences (^*^P ≤ 0.05; ^**^P ≤ 0.01; ^***^P ≤ 0.001).

In agreement, we further investigated the effect of *CsPIF1* on Thea content from transient knock-down *CsPIF1* expression in tea plants, conducting antisense oligonucleotide (asODN) technology (Fig. 7A). Consequently, compared with control treatment (sense oligonucleotide, sODN), the expression of *CsPIF1* in the tender shoots significantly decreased with asODN treatment both in ‘LJ43’ (*P* < 0.001) and ‘BJG’ (*P* < 0.001) (Fig. 7B) alongside remarkable increase of Thea content (*P* < 0.01 and *P* < 0.05, respectively). These results above aroused our interest to explore the genes which regulate Thea synthesis and transport respond to knock-down *CsPIF1* expression, and thus we analysed the expression levels of the selected DEGs using the asODN treatment materials above.

First, the *CsCAT1*, *CsAAP7*, and *CsAAP3* involved in the regulation of Thea transport were significantly upregulated by knockdown *CsPIF1* expression in the new shoots of tea plants (Fig. 7C and D). Meanwhile, *CsGOGAT*, *CsGDH*, *CsTS*, and *CsAlaDC* play considerable roles in the Thea synthesis and also showed significant increase in expression, especially in the case of *CsGOGAT*, which was identified to have a nonsynonymous SNP179068992 regulating Thea content. At the same time, we found *CsAlaAT2*, which can catalyze L-alanine and 2-oxoglutalate to pyruvate and L-glutamate [41], also with upregulation expression. Taken together, these results indicated that *CsPIF1* negatively influenced Thea content by effecting vitally functional genes associated with Thea biosynthesis and transport. Moreover, CsPIF1 protein was fused at the C-terminus to GFP and transiently expressed in *Nicotiana benthamiana* to observe its subcellular localization and obtain insight into the molecular function. Consequently, the signal of GFP-CsPIF1 in compartments was localized throughout the nucleus (Fig. 7E).

### Identification of *CsPIF1* regulating theanine accumulation in *Arabidopsis* fed with ethylamine

**Figure 8:**
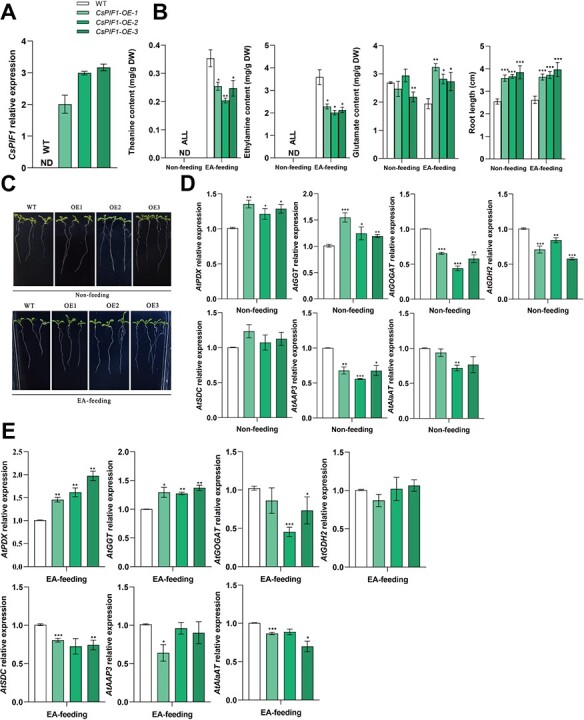
Phenotypes, determination of theanine and its synthesis precursors contents, and related genes expression in *CsPIF1* transgenic wild type lines (*CsPIF1-OE1, CsPIF1-OE2,* and *CsPIF1-OE3*). **A***CsPIF1* expression in three *CsPIF1* transgenic wild type lines, as compared with negative control WT lines. *CsPIF1* overexpressed in *Arabidopsis* driven by a 35S promoter. ND, not detected. **B** Theanine, glutamate, ethylamine contents, and root length were determined in these *Arabidopsis* lines. **C** The phenotype of seven-day-old seedlings of wild type, and transgenic lines in 1/2MS medium supplemented with or without 10 mM ethylamine. **D** and **E** The homologous genes expression related to theanine biosynthesis, transport and hydrolysis in these *Arabidopsis* lines supplemented with or without 10 mM ethylamine. Data are means ± SEs from at least three independent transgenic lines with three biological replicates. DW, dry weight. Asterisks above the error bar indicate significant differences (^*^P ≤ 0.05; ^**^P ≤ 0.01; ^***^P ≤ 0.001).

Based on the validation carried out in this study above, we finally identified the function of *CsPIF1* on Thea accumulation in *Arabidopsis* fed with ethylamine (EA). Free amino acid compositions profiling indicated that the Thea content degraded in three independent *CsPIF1*-overexpressing *Arabidopsis* plants (*CsPIF1-OE1, CsPIF1-OE2,* and *CsPIF1-OE3*) (Fig. 8A) grown on the half-strength MS medium with 10 mM EA chloride, as well as longer root length compared with wild type (WT) (Fig. 8B and C), which was consistent with the inhibitory effects of Thea on root growth [42]. At the same condition, another substrate Glu in three *CsPIF1*-overexpression lines was significantly higher than in the WT, suggesting that the EA availability is the key factor for the Thea accumulation (Fig. 8B). Moreover, we found that the *Arabidopsis pif1* mutant (Fig. S8, see online supplementary material) accumulated higher levels of Thea synthesis precursors Glu and Ala no matter whether the whole plant or in leaves than WT seedlings. Strikingly, Gln, the analog of the Thea, also had a higher amount in *pif1* (Fig. S8, see online supplementary material). These results further indicated that *CsPIF1* is negatively correlated with Thea accumulation in *Arabidopsis* fed with EA.

We further validated the homologous genes expression of these tea plant DEGs above in these *Arabidopsis* lines. It is not unexpected that among them, the expression of *AtGOGAT*, *AtGDH2*, and *AtAAP3* in *CsPIF1-OE1*, *CsPIF1-OE2*, and *CsPIF1-OE3* were all significantly downregulated compared to their WT (Fig. 8D). Moreover, we observed that there was no significant increase, even with the notable downward trend when fed with EA (Fig. 8E). Meanwhile, both *AtSDC*, which is a homologous gene of *CsAlaDC* in *Arabidopsis*, and *AtAlaAT* exhibited a nonsignificant increase in *CsPIF1*-overexpression lines following positive decline supplemented with EA. The range of values seen for the expression of key genes related to Thea biosynthesis and transport were not upregulated upon precursor EA feeding. Moreover, the *AtPDX* and *AtGGT* expression, which are the homologous genes of *CsPDX* and *CsGGT* reported to be able to hydrolyze the Thea [43, 44], exhibited a notable upward trend supplemented with or without EA. Taken together, these results allow us to conclude that *CsPIF1* negatively regulates the Thea content by mainly co-effecting the expression of genes involved in the regulation of Thea biosynthesis, transport, and hydrolysis in tea plants.

## Discussion

### Free amino acids distribution among different varieties and negative patterns with the level of chlorophyll

Increasing the taste of tea fusion and improving its nutritional quality are of both economic and social interest. Therefore, in this study great efforts have been devoted to the collection and evaluation of tea accessions following FAA analysis. Previous research on this topic largely focused on special mutation varieties to reveal insights into differential accumulation of FAAs, such as ‘Huabai 1’ [45] and ‘Yinghong 9’ [23]. In this study, we analysed a wide range of tea accessions from the main tea-producing regions. A novel phenomenon has been found: that the FAA level of wild relatives mainly from the origin area of Yunnan were significantly lower than that of the three varieties of *C. sinensis*, reflecting the different shifts of underlying metabolites [46]. Among intraspecific accessions, for mean contents of arginine (Arg) and glutamine (Gln), there were significant differences in the *sinensis* and *pubilimba*, *sinensis* and *assamica* varieties. Meanwhile, *sinensis* displayed significant difference with *assamica* and *pubilimba* varieties in terms of alanine (Ala) and the diversity index helps us with the feasibility and diversity of FAAs of tea germplasms from the main tea-producing regions. Considering that, these results could be the intensive evidence that allows us to clarify the molecular mechanism for biosynthesis, transport, and hydrolysis pathway of these compositions from the genetic perspective in the following GWAS study [47].

Furthermore, correlation analysis of metabolites validated that there were significantly negative patterns between the levels of chlorophyll and FAAs. A previous study reported that production and transportation of photosynthetic products could be inhibited when magnesium is absent [48] and Zhang *et al*. have found higher amounts of amino acid accumulation in tea roots under magnesium deficiency condition compared with normal magnesium levels [49]. Meanwhile, magnesium participates in the synthesis of chlorophyll in plant cells [50] and the supplementation of magnesium could increase the amino acid level both in tea roots and shoots [49]. Therefore, an important finding of this work is that the inhibition of chloroplast development led to related genes expression and protein abundance change mainly following FAA accumulation by cell ultrastructure, metabolites, and transcriptome analysis. Moreover, these shifts are not surprising, given that most leaf protein is housed within the chloroplast where chlorophyll a/b and Rubisco binding protein are the largest reservoirs of recoverable nitrogen, indicating the potential balance mechanism of carbon and nitrogen metabolism, and recruit other genes and protein for survival following the degradation of chloroplasts [51–53]. Therefore, the results in this study indicated that the chlorophyll abundance can be measured to evaluate FAA amounts in tea plants (Fig. 3B).

### Validation of a nonsynonymous SNP and the negative regulation of *CsPIF1* on theanine content

Among these associations, we have further demonstrated the power of WGCNA from transcription level based on the FAA trait for mining candidate genes to dissect complex phenotypes. First, among the differentially expressed genes (DEGs), *CsCAT1* and *CsVAR* showed no significant correlation with FAAs on WGCNA analysis (Table S2, see online supplementary material). While we identified *CsPIF1* combined with our previous QTL mapping results [32] which have high Pearson correlation coefficients (PCC > 0.95), showing a negative relationship with theanine (Thea) by expression analysis among different cultivars and different tissues and shade treatment (Fig. 5B, F and G). *CsPIF1* was recently reported to protect tea plants from tea geometrid larvae by regulating the synthesis and release of benzyl nitrile [54]. Here, we found the increased Thea content was significantly induced when *CsPIF1* expression was knocked down in the new shoots of tea plants (Fig. 7A and B) and we validated this association achieved by regulating key functional genes involved in the regulation of Thea biosynthesis (*CsGOGAT*, *CsGDH*, *CsTS1*, and *CsAlaDC*), and transport (*CsCAT1*, *CsAAP7*, and *CsAAP3*) (Fig. 7C and D). In addition, due to the difficulty of resource collection and preservation, single nucleotide polymorphism (SNP) identification based on functional genes is still in early stages and with the continuous in-depth research on the genome of tea plants, it helps us further understand the impact of genetic variations on the tea quality and breeding [55]. In this study, we validated a nonsynonymous SNP (SNP179068992) within coding sequences of the *CsGOGAT* using KASP, with three specific genotypes (CC, TC, and TT) (Fig. 6A and Table 3). In detail, genotype CC was the dominant genotype at the SNP179068992 site based on 148 tea accessions and significantly different with TT, which accumulated substantially more Thea among three genotypes (Table S3, see online supplementary material). Therefore, SNP179068992 could be utilized for distinguishing the Thea characteristic of tea plants (Fig. 6B).

Moreover, the *CsPIF1*-overexpressing *Arabidopsis* plants confirmed that the degradation of Thea with ethylamine (EA) concentrations (Fig. 8B) and the longer root length compared with wild type (WT) (Fig. 8B and C) because the absorption of EA fed was far less than that of WT, although another substrate glutamate (Glu) accumulation in the *CsPIF1*-overexpression lines was significantly higher than WT (Fig. 8B). Moreover, the expression analysis of *CsGOGAT* with a nonsynonymous SNP showed significant increase in transient knock-down *CsPIF1* expression and the homologous gene *AtGOGAT* was significantly downregulated in these overexpressing *Arabidopsis* lines compared to their WT with or without EA feeding (Fig. 8D). Meanwhile, the *Arabidopsis pif1* mutant also accumulated higher levels of Thea synthesis precursors Glu and Ala no matter whether it was the whole plant or in leaves than WT seedlings (Fig. S8, see online supplementary material) and CsPIF1 was located in the nucleus (Fig. 7E), consistent with CsGS1 and CsGS2 subcellular localization results [56]. These results suggested that *CsPIF1* acts as a negative regulator of Thea content by effecting the vitally functional genes expression related to Thea biosynthesis in tea plants. What is more, the overexpressing *Arabidopsis* lines were analysed to exhibit the notable upward tendency of *AtPDX* and *AtGGT* expression supplemented with or without EA (Fig. 8E). Together, these results allow us to conclude that the negative regulation of Thea by *CsPIF1* is also associated with hydrolysis genes in tea plants. Therefore, these results offer useful approaches for dissection of complex composite traits in tea plants.

## Conclusion

The FAA trait of tea plants is a relatively complex quantitative trait. By analysing the variation of FAAs among 339 tea accessions in two years, we can know that the determined components of FAAs including arginine, glutamine, glutamate, alanine, and theanine with the highest diversity index (2.03) differed among different genetic resources and intraspecific accessions. Meanwhile, their amounts in *C. sinensis* were all significantly higher than in the wild relatives. Our study confirmed the significant opposite trend between chlorophyll and FAA profiles. This finding was genetically validated by following cell ultrastructure results that samples possessing lower FAA levels had the abundant chloroplasts and tightly stacked grana. In gene expression analysis of different cultivars and tissues, and transient knock-down expression experiments in tea plants, *CsPIF1* has been identified to negatively regulate theanine accumulation, consistent with its response to shade results. A further important finding concerns the association between *CsPIF1* and functional genes associated with theanine synthesis (*CsGDH*, *CsTS*, *CsGOGAT*, and *CsAlaDC*), transport (*CsCAT1*, *CsAAP7*, and *CsAAP3*), whose transcription significantly increased under knockdown *CsPIF1* expression, especially *CsGOGAT* in which we identified a nonsynonymous SNP. These findings were in agreement with the heterologous expression of *CsPIF1* in *Arabidopsis*, especially under ethylamine application. Therefore, these results offer insights and benefits for the conservation, evaluation, and utilization of tea germplasms, achieving the ultimate goal of tea plants’ genetic improvement and breeding, and it provides valuable information and approaches for further deciphering complex composite traits in tea plants.

## Materials and methods

### Plant materials and experimental design

A previously assembled natural diversity of tea accessions was used to study catechin metabolites [57]. As such, to obtain reliable data for FAAs and genetic analysis, a total of 339 tea accessions were sampled, currently preserved as genetic resources in the National Tea Germplasm Repository at Hangzhou of TRICAAS, China under the same cultivation conditions and agricultural practices. In detail, the manifold distribution and variation including 220 accessions of *C. sinensis* (L.) O. Kuntze var. *sinensis* (*sinensis* variety); 58 accessions of *C. sinensis var. assamica* (*assamica* variety); 25 accessions of *C. sinensis* var. *pubilimba* Chang (*pubilimba* variety); five accessions *C.* sp. (uncategorized germplasms), 29 wild tea relatives, as well as two *Camellia* L. non-*Thea* section species, *C. reticulata* Lindl. and *C. oleifera* Abel are shown in Table S4 (see online supplementary material). Among them, 22 accessions with FAA differences were selected for further cell ultrastructure, chlorophyll amount, metabolites profiling, and transcriptomic analysis. In agreement, three biological replicates were used for analysis in ‘two and a bud’ (one bud with two tender leaves) collected in the first round from March to April, 2021, respectively, and the *Arabidopsis pif1* mutant was from Xingwang Deng Lab, in the Southern University of Science and Technology, China.

The healthy 1-year rooted cuttings of elite green cultivar ‘Longjing 43’ (‘LJ43’) and distinctive albino cultivar ‘Baijiguan’ (‘BJG’), the parents contributing the transgressive segregation among the F1 population, were conducted under the same shade conditions by covering with a black cloth when tender shoots reached the ‘two and a bud’ stage with six pots per treatment. The pots had a 30 cm outer diameter with 2 cm × 2 cm drainage holes in the base. Afterwards, processed samples were collected on 2 April, 9 April, 5 May, and 12 May, respectively, and were divided into two parts. The six biological replicates had FAA amounts determined, and another six biological duplicates were quickly fixed with liquid nitrogen following storage in a −80°C refrigerator until gene expression analysis. The *Arabidopsis* seed was vernalized at 4°C for 3 d following transfer into an incubator for 15 days. The condition set was 25°C / 21°C for 16 h / 8 h day / night with 60% relative humidity.

### Free amino acids and chlorophyll determination among tea accessions

Two hundred milligrams of fine tea powder were extracted with 10 mL of boiling water for 30 minutes, with intermittent shaking every 10 minutes, and centrifuged at 3500 r/min for 10 minutes. The supernatants were filtered through a 0.22-μm filter membrane and then performed according to Waters AccQ-Tag Ultra Derivatization Kit (Waters Co., Milford, MA, USA). ACQUITY UPLC H-Class system (Waters Co., Milford, MA, USA) with ACCQ-TAG™ ULTRA C18 column (1.7 μm, 2.10 × 100 mm, Column) was utilized for analysis. The flow rate was 0.7 mL/min with mobile phases purchased from Waters Corp. (Milford, MA, USA) and the gradient program was as follows: 0–7.99 min, linear gradient from 10% to 4.0% A; from 90% to 36.3% C; from 0% to 59.7% D; 7.99–10.20 min, back to 10% A; linear gradient to 90% C; linear gradient to 0% D. Chromatographic peaks were identified by UV spectra using diode array detector, and retention times were compared with amino acid standards.

Meanwhile, fresh samples were finely minced with liquid nitrogen, following 0.1 g ground mixed with 95% ethanol and incubated in darkness for 12 h. The extracts were determined using ultraviolet spectrophotometry with three independent biological replicates. Total chlorophyll (Chl), chlorophyll *a* (Chl a), and chlorophyll *b* (Chl b) were analysed at 665 nm and 649 nm, respectively.

### Sample extraction and metabolites profiling based on LC–MS

Three biological samples to fine powders using a mixer mill (MM 400, Retsch) with a zirconia bead for 1.5 min at 30 Hz after freeze-drying by vacuum freeze-dryer (Scientz-100F). One hundred milligrams powder were dissolved with 1.2 mL 70% methanol solution overnight at 4°C, vortex 30 seconds every 30 minutes for six times in total, following centrifugation at 12000 rpm for 10 min.

The extracts were conducted using an UPLC-ESI-MS/MS system (UPLC, SHIMADZU Nexera X2, USA; MS, Applied Biosystems 6500 Q TRAP, USA) with Agilent SB-C18 (1.8 μm, 2.10 × 100 mm). The mobile phases were composed of pure water with 0.1% formic acid (solvent A) and acetonitrile with 0.1% formic acid (solvent B). The measurements gradient evolution program was as follows: 0.00 min, 5% B; 0.00–9.00 min, 95% B; 10.00–11.10 min, 5% B, with 0.35 mL flow rate per minute and 40°C column temperature. The condition parameters of ESI source were as follows: temperature 550°C; gas II, curtain gas, and ion source gas I were set at 60, 25, and 50 psi, respectively; ion spray voltage was 5500 V/−4500 V.

### Transmission electron microscopy analysis

The slices volume of fresh cells (1 mm × 1 mm × 1 mm) were put in the electron microscope fixative at room temperature for 2 h, and then transferred to the 4°C refrigerator, rinsed with 0.1 M sodium phosphate buffer PB (pH 7.4) for three times and 15 minutes each time following fixed with 1% OsO_4_ in 0. 1 M PB (pH 7.4). After fixation, the samples were dehydrated with an ethanol gradient for penetration according to the gradient: acetone: 812 embedment agents = 3 : 1 for 2–4 h; acetone: 812 embedment agents = 1 : 1 penetration overnight; acetone: 812 embedment agents = 1 : 3 for 2–4 h. Subsequently, the samples were inserted into the embedment plate at 37°C overnight. These materials were then embedded with polymerization in 60°C for 48 h, slicing 60–80 nm semithin sections with an ultramicrotome (Leica UC7, Leica Microsystems, Wetzlar, Germany). For further analysis, the slices were observed using a transmission electron microscope (Thermo Scientific™ Talos L120C, USA) after double staining with uranium acetate saturated alcohol solution and Lead citrate avoid CO_2_ staining.

### RNA sequencing and gene expression profiling using RT-qPCR

Total RNA was extracted using the RNAprep Pure Plant Kit (Tiangen Biotechnology, Beijing, China) according to the manufacturer’s recommendations. The mRNA with PolyA structure was enriched by Oligo (DT) magnetic beads, interrupting to a fragment with a length of around 300 bp by ion interruption and the first-strand cDNA was synthesized by random primers and reverse transcriptase. Afterward, second-strand cDNA was subsequently generated using the Illumina TruSeq RNA Sample Prep Kit (Illumina, Inc., San Diego, CA, USA), quantifying with a Bio-Rad KIT iQ SYBR Green kit (Bio-Rad CFX 96, Bio-Rad Laboratories, Inc., Hercules, CA, USA). The cDNA constructed with an insert fragment size of 450 bp, were assessed with an Agilent 2100 Bioanalyzer (Agilent Technologies, Santa Clara, CA, USA), and sequenced by the Illumina HiSeq 2000 system with paired-end 225-bp reads. 3′ Adapters and low-quality raw reads with average quality score lower than Q20 were trimmed by Cutadapt and FastQC (https://www.bioinformatics.babraham.ac.uk/projects/fastqc/) software in accordance with default parameters.

The clean reads were subsequently aligned to the *C. sinensis var. sinensis* genome [36] using HISAT2 (http://ccb.jhu.edu/software/hisat2/index.shtml) with BWT algorithm. qRT-PCR was conducted for validation of gene expression as described previously [32] with the PrimeScriptTM^RT^ Reagent Kit (TaKaRa). The relative expression was calculated using the 2^−ΔΔCt^ method and specific primers are listed in Table S5 (see online supplementary material).

### Subcellular localization through transient expression in tobacco and suppression analysis in tea shoots

The double enzyme digestion products of *CsPIF1* plasmid were transferred into pBWA(V)HS-GFP through T4 ligase, and the DNA link protocol: nuclease-free water 5.5 μL, 10 $\times$ buffer 1 μL, vector 1 μL, product 2.5 μL at 20°C for 3 h. The resultant vectors were subsequently transformed into Agrobacterium GV3101 competent cells cultured at 30°C for 2 d, following monoclonal positive identification. Then a solution with OD600 value of 0.6 was transiently introduced into *N. benthamiana* leaves by injection following being held for 2 days at 25°C in the dark with an empty vector as the control. The *N. benthamiana* leaves and were examined using a Nikon C2-ER Laser confocal microscope (Japan).

The antisense oligonucleotides (asODNs) of *CsPIF1* were designed according to Soligo software: http://sfold.wadsworth.org/cgi-bin/index.pl. The tender ‘one and a bud’ (one bud with one tender leave) were plucked from ‘LJ43’ and ‘BJG’ with at least six biological replicates and incubated with 20 μM asODN solution, sense oligonucleotides (sODN) as control. The tender shoot tips were sampled to analyse the FAA level and genes expression after being incubated for one week.

### Stable transformation verification of *CsPIF1* function

For *CsPIF1* function verification, the coding sequence of *CsPIF1* was amplified via PCR and cloned into the pQB-V3 vector. Then the product was recombined into the pk7FWG2.0 vector containing the 35S promoter using the Gateway (Invitrogen) technology [58]. The resulting construct was electroporated into *Agrobacterium tumefaciens* GV3101 and used to infect the *Arabidopsis* Col-0 according to the standard flower dipping method [59]. The T2 transgenic lines were verified by PCR verification and homozygous T3 was used for further analysis at the seedling stage. *CsPIF1-OE Arabidopsis* lines and Col-0 were germinated on half-strength MS medium with or without 10 mM ethylamine chloride after surface sterilization incubating in growth chambers under a 16-h light / 8-h dark photoperiod at 21°C and 23°C, respectively. After 15 d of growth, seedlings were harvested for FAAs determination using the method described above, with at least three replicates containing 15–20 seedlings.

### SNP mining and identification

The DEGs that had an FPKM >10 were responsible for theanine (Thea) metabolism aligned to the genome for SNP screening. Filtering sequencing reads with SNP base quality was more than 99%, the number of sites covering reads and supporting mutation exceeding 8$\times$ and 2$\times$, respectively. The *P*-value of SNP <0.01. A total of 148 tea accessions were collected for DNA extraction, following a DNAsecure Plant Kit (Tiangen Biotech Co., Ltd, China), diluting for genotyping in subsequent experiments. The SNPs coding Thea genes were tested by Kompetitive Allele-Specific PCR (KASP) based on Douglas array tape (Laboratory of the Government Chemist, UK). Afterwards, genotype data were obtained using intelliscore software. The functional investigation primers and SNPs for KASP are displayed in Table S3 (see online supplementary material).

### Statistical analysis

The FAAs and chlorophyll amounts were calculated for each trait using by Excel, IBM SPSS Statistics version 26.0 (SPSS Inc., Chicago, IL, USA) statistical software, comparing differences among means with analysis of variance (ANOVA) and Tukey’s test at a threshold of *P* < 0.05. Principal component analysis (PCA) was performed by statistics function prcomp within R (www.r-project.org).

The metabolite raw files of samples were analysed qualitatively and quantitatively by mass spectrometry, using Analyst 1.6.3 software. The characteristic ions of each substance were screened through the triple quadrupole, and the signal intensities of the characteristic ions were obtained in the detector. The peak area of each chromatographic peak was integrated and corrected with integrity and correct using multiquant software. Significantly regulated metabolites from different groups were determined by Variable Importance in Projection ≥1 and absolute log_2_FC (fold change) ≥1 with R package MetaboAnalystR. Genes expression and network analysis were imported into Cytoscape (1991, 1999 Free Software Foundation, Inc.) for visualizing.

## Acknowledgements

Thanks to Professors Xingwang Deng and Hua Zhou (Southern University of Science and Technology, China) for kindly providing the seeds of the *Arabidopsis pif1* mutant. This work was supported by the National Natural Science Foundation of China (32072631, U19A2030), the China Agricultural Research System of MOF and MARA (CARS-019), and the Chinese Academy of Agricultural Sciences through the Agricultural Science and Technology Innovation Program (CAAS-ASTIP-2021-TRICAAS) to LC. 

## Author contributions

L.C. and D.N. conceived and supervised this study; R.H., Z.W., S.Z., and F.L. performed the experiments; L.C. and R.H. designed the research and analysed the data; W.W., M.Y., and H. L. provided suggestions on the manuscript; R.H. and L.C. wrote and revised the manuscript.

## Data availability

All the data in this study are provided in the article and its supplementary file.

## Conflict of interest statement

The authors declare that they have no conflict of interest.

## Supplementary data

Supplementary data is available at *Horticulture Research* online.

## Supplementary Material

Web_Material_uhad263
