# Supplementary material for: Comprehensive dissection of variation and accumulation of free amino acids in tea accessions
Source: Hortic Res. 2023 Dec 13;11(1):uhad263. doi: 10.1093/hr/uhad263 (PMC10833077; doi:10.1093/hr/uhad263)
Supplement: Web_Material_uhad263 [file web_material_uhad263.zip › Supplementary Figures.pdf]

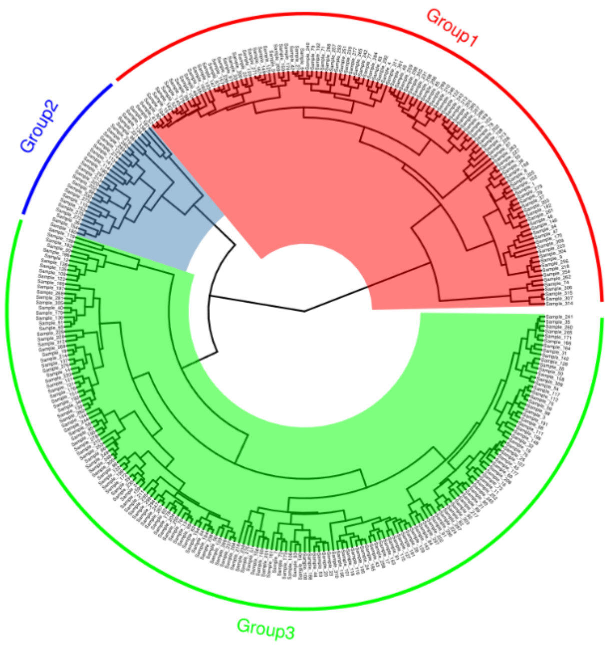

Figure S1. Group distribution of 339 tea accessions for free amino acids content.

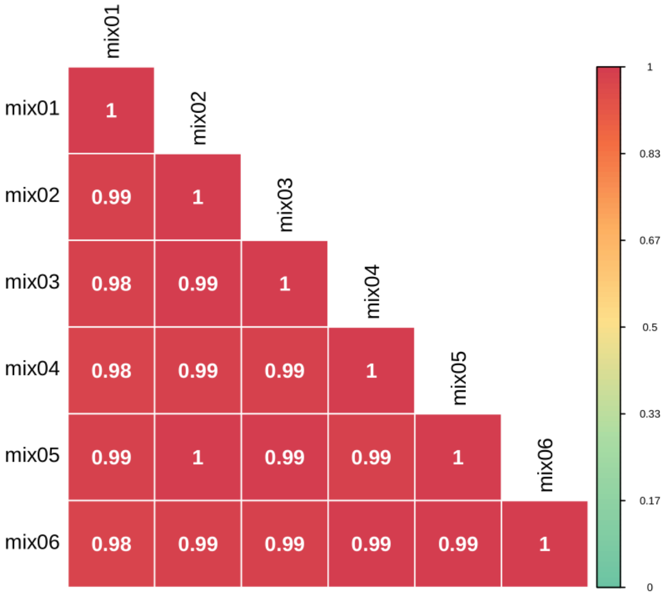

Figure S2. Correlation between metabolites.

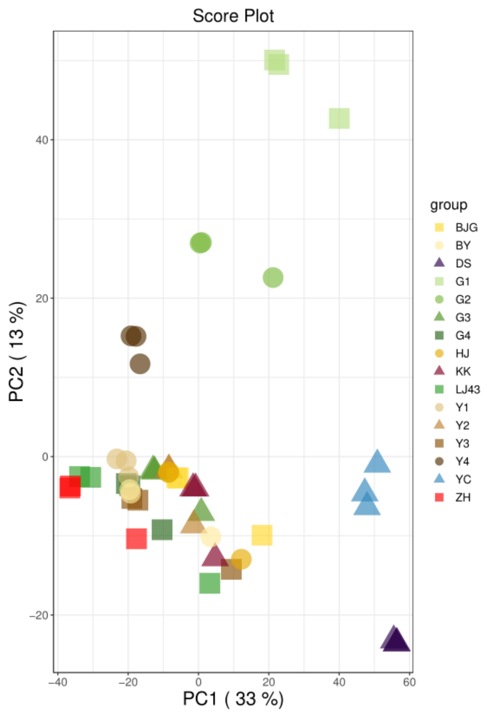

Figure S3. Principal component analysis at transcription level for parents and offspring. G1-G4 and Y1-Y4: green and yellow F1 offspring from amino acids artificial segregating population of *Camellia sinensis* ‘Longjing 43’ (‘LJ43’) × *C. sinensis* ‘Baijiguan’ (‘BJG’); BY: *C. sinensis* ‘Baiye 1’; DS: *C. reticulata* Lindl.; HJ: *C. sinensis* ‘Huangjingya’; KK: *C. sinensis* var. *pubilimba* ‘Kekecha’; YC: *C. oleifera* Abel; ZH: *C. sinensis* ‘Zhonghuang 2’.

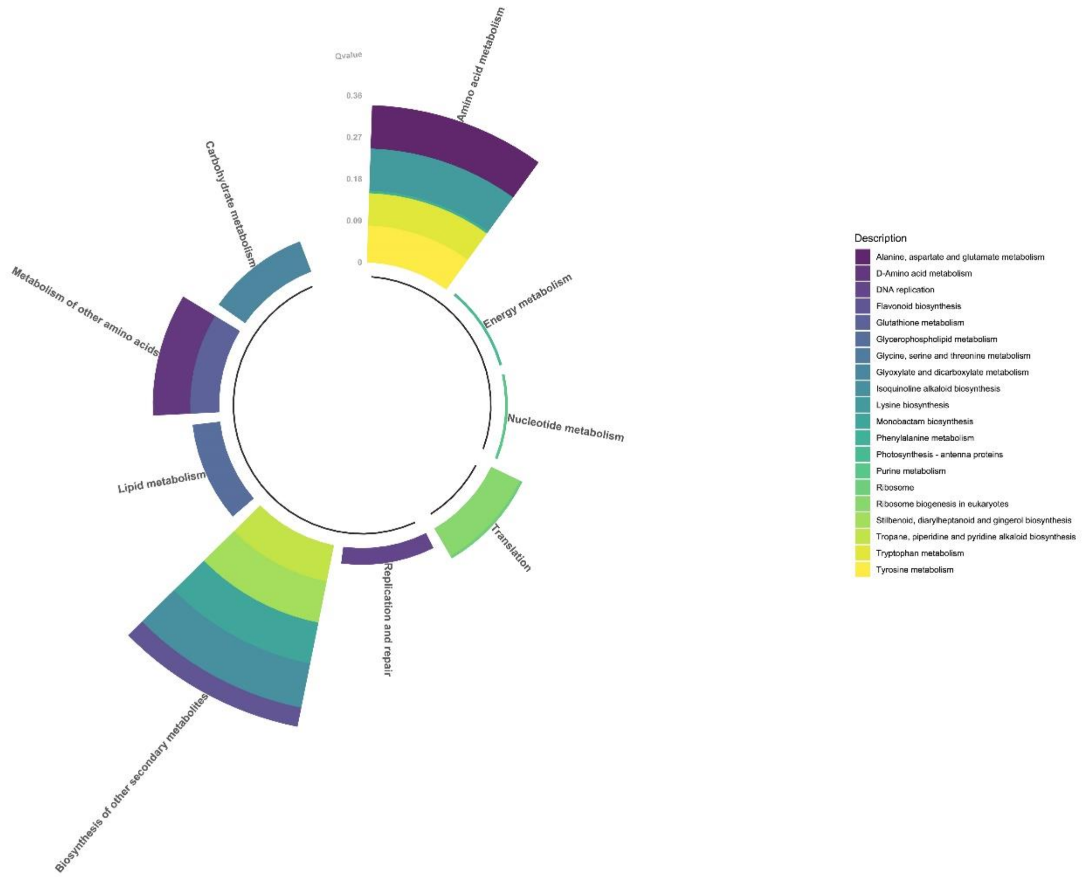

Figure S4. KEGG analysis for Top 20 enriched KEGG pathways among DEGs in ‘BY’ and ‘YC’ comparison.

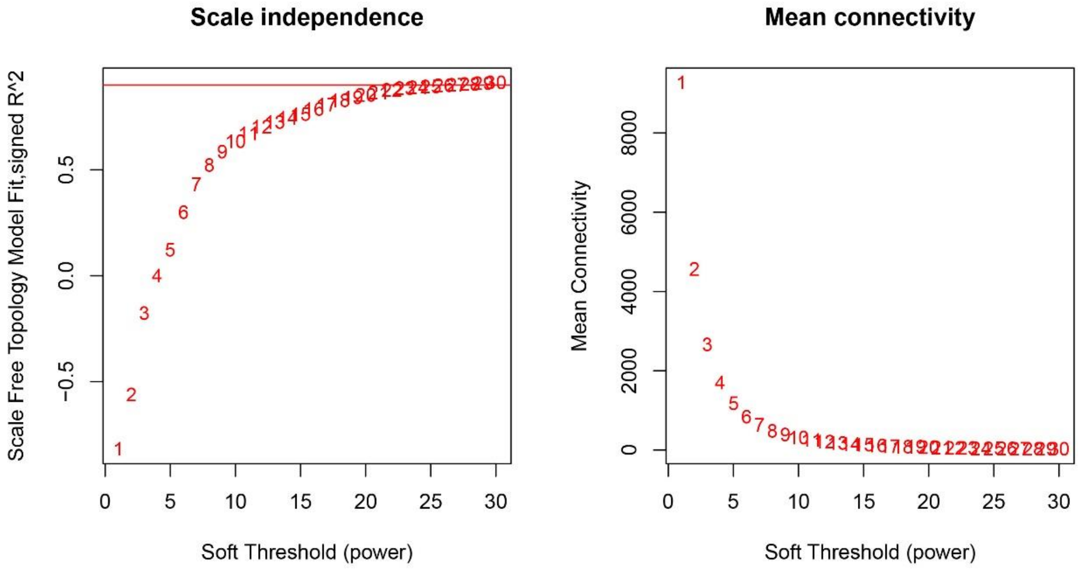

Figure S5. Threshold parameters.

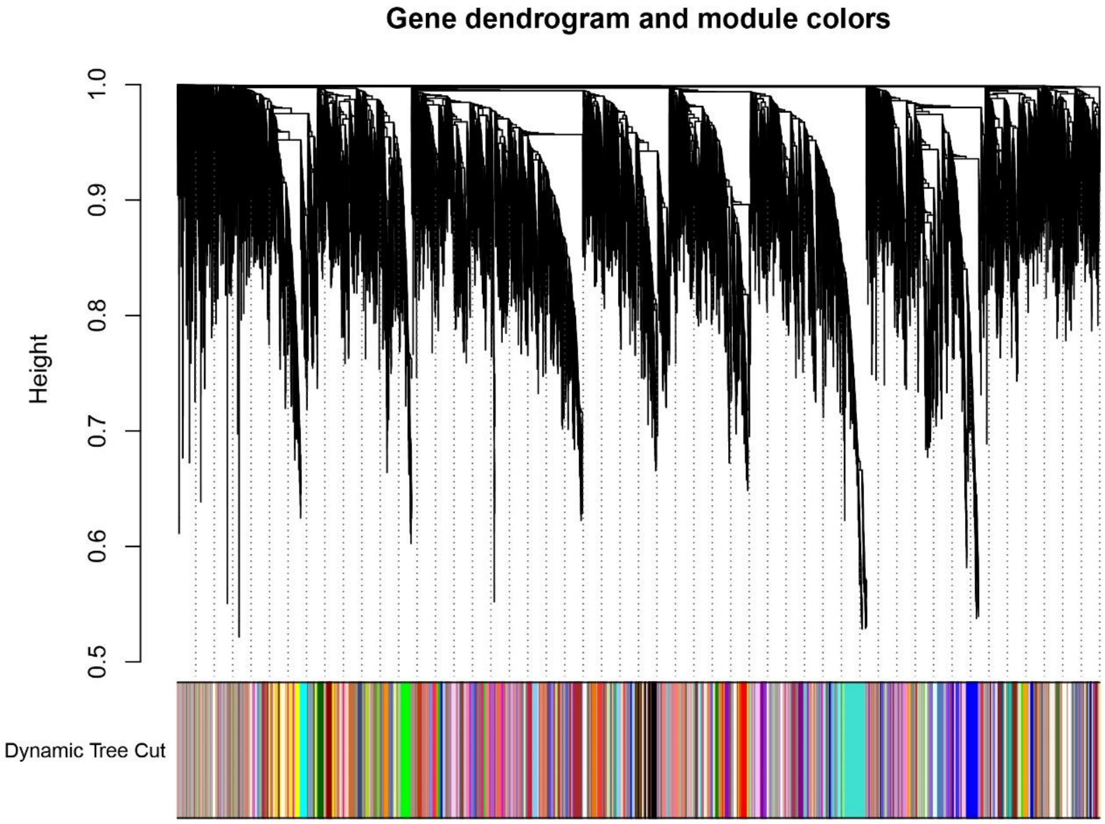

Figure S6. Similar modules merging.

```
SNP179068992_1.seq   MSVISNSVVQTRNNNNNSVIPSITKPFVALNRVRITKAFVDKRSKTKSNVLENRFCGTKLRACERLHLWQSDGPGLDPKL     80
SNP179068992_2.seq   MSVISNSVVQTRNNNNNSVIPSITKPFVALNRVRITKAFVDKRSKTKSNVLENRFCGTKLRACERLHLWQSDGPGLDPKL     80

SNP179068992_1.seq   KVVVRSALSQVPEKPLGLYDPSFDKDSCGVGFVAELSGESSRKTVTDAVEMLIRMSHRGACGCETNTGDGAGILVALPHQ    160
SNP179068992_2.seq   KVVVRSALSQVPEKPLGLYDPSFDKDSCGVGFVAELSGESSRKTVTDAVEMLIRMSHRGACGCETNTGDGAGILVALPHQ    160

SNP179068992_1.seq   FYKEVAKDVGFELPPPGEYAVGMFFLPTSENRRAQSKIVFTKVAESLGHTVLGWRTVPTDNSGLGNSALQTEPVIEQVFL    240
SNP179068992_2.seq   FYKEVAKDVGFELPPPGEYAVGMFFLPTSENRRAQSKIVFTKVAESLGHTVLGWRTVPTDNSGLGNSALQTEPVIEQVFL    240

SNP179068992_1.seq   TPTPKSKVDFEQQLYILRRVSMVAIRAALNLQHGGVRDFYICSLSSRTVVYKGQLKPNQLKQYYYADLGNERFTSYMALI    320
SNP179068992_2.seq   TPTPKSKVDFEQQLYILRRVSMVAIRAALNLQHGGVRDFYICSLSSRTVVYKGQLKPNQLKQYYYADLGNERFTSYMALI    320

SNP179068992_1.seq   HSRFSTNTFPSWDRAQPMRVLGHNGEINTLRGNVNWMKAREGLLKCKELGLSKNEMKKLLPIVDASSSDSGAFDGVLELL    400
SNP179068992_2.seq   HSRFSTNTFPSWDRAQPMRVLGHNGEINTLRGNVNWMKAREGLLKCKELGLSKNEMKKLLPIVDASSSDSGAFDGVLELL    400

SNP179068992_1.seq   VRAGRSLPEAVMMMIPEAWQNDKNMDPHRKALYEYFSALMEPWDGPALISFTDGRYLGATLDRNGLRPGRFYVTHSGRVI    480
SNP179068992_2.seq   VRAGRSLPEAVMMMIPEAWQNDKNMDPHRKALYEYFSALMEPWDGPALISFTDGRYLGATLDRNGLRPGRFYVTHSGRVI    480

SNP179068992_1.seq   MASEVGVVDIQPEDVCRKGRLNPGMMLLVDFEKHVVVDDEELKQQYSLSRPYGEWLKRRKIELKDIVESVSKSQRVPQAI    560
SNP179068992_2.seq   MASEVGVVDIQPEDVCRKGRLNPGMMLLVDFEKHVVVDDEELKQQYSLSRPYGEWLKRRKIELKDIVESVSKSQRVPQAI    560

SNP179068992_1.seq   AGVLPASNDDDNMENMGIHGLLAPLKTFGYTVESLEMLLLPMAKDGVESLGSMGNDAPLAVMSNREKLTFEYFKQMFAQV    640
SNP179068992_2.seq   AGVLPASNDDDNMENMGIHGLLAPLKTFGYTVESLEMLLLPMAKDGVESLGSMGNDAPLAVMSNREKLTFEYFKQMFAQV    640

SNP179068992_1.seq   TNPPIDPIREKIVTSMECMIGPEGDLTETTEEQCHRLSLKGPLLSIEEMEAIKKMNYRGWRSKVLDITFSKDRGSKGLEE    720
SNP179068992_2.seq   TNPPIDPIREKIVTSMECMIGPEGDLTETTEEQCHRLSLKGPLLSIEEMEAIKKMNYRGWRSKVLDITFSKDRGSKGLEE    720

SNP179068992_1.seq   TLDRICAEAHDAIKEGYKTLVLSDGAFSSKRVAVSSLLAVGAVHHHLVKKLERTRIGLIVESAEPREVHHFCTLVGFGAD    800
SNP179068992_2.seq   TLDRICAEAHDAIKEGYKTLVLSDGAFSSKRVAVSSLLAVGAVHHHLVKKLERTRIGLIVESAEPREVHHFCTLVGFGAD    800

SNP179068992_1.seq   AICPYLAIEAIWRLQVDGKIPPKASGEFQSKDELIKKYFKASNYGMMKVLAKMGISTLASYKGAQIFEAVGLSSEVMERC    880
SNP179068992_2.seq   AICPYLAIEAIWRLQVDGKIPPKASGEFQSKDELIKKYFKASNYGMMKVLAKMGISTLASYKGAQIFEAVGLSSEVMERC    880

SNP179068992_1.seq   FAGTPSRVEGATFEALANDVLQLHDLAFPTRVFPPGSAEAVSLPNPGDYHWRKGGEVHLNDPLAISKLQEAARVNSVAAY    960
SNP179068992_2.seq   FAGTPSRVEGATFEALANDVLQLHDLAFPTRVFPPGSAEAVSLPNPGDYHWRKGGEVHLNDPLAISKLQEAARVNSVAAY    960

SNP179068992_1.seq   KEYSKRIQELNKNCNLRGLLKFIEAEVKVPLDEVEPASEIVKRFCTGAMSYGSISLEAHTTLAMAMNKIGGKSNTGEGGE   1040
SNP179068992_2.seq   KEYSKRIQELNKNCNLRGLLKFIEAEVKVPLDEVEPASEIVKRFCTGAMSYGSISSEAHTTLAMAMNKIGGKSNTGEGGE   1040

SNP179068992_1.seq   NPSRMEPLPNGSMNPKRSAIKQVASGRFGVSSYYLTNADELQIKMAQGAKPGEGGELPGHKVIGDIAITRNSTAGVGLIS   1120
SNP179068992_2.seq   NPSRMEPLPNGSMNPKRSAIKQVASGRFGVSSYYLTNADELQIKMAQGAKPGEGGELPGHKVIGDIAITRNSTAGVGLIS   1120

SNP179068992_1.seq   PPPHHDIYSIEDLAQLIHDLKNANPGARISVKLVSEAGVGVIASGVVKGHADHVLISGHDGGTGASRWTGIKSAGLPWEL   1200
SNP179068992_2.seq   PPPHHDIYSIEDLAQLIHDLKNANPGARISVKLVSEAGVGVIASGVVKGHADHVLISGHDGGTGASRWTGIKSAGLPWEL   1200

SNP179068992_1.seq   GLAETHQTLVANDLRGRTVLQTDGQLKTGRDVAIAALLGAEEFGFSTAPLITLGCIMMRKCHKNTCPVGIATQDPVLREK   1280
SNP179068992_2.seq   GLAETHQTLVANDLRGRTVLQTDGQLKTGRDVAIAALLGAEEFGFSTAPLITLGCIMMRKCHKNTCPVGIATQDPVLREK   1280

SNP179068992_1.seq   FAGEPEHVINFFFMLAEEVREIMSQLGFRTINEMVGRSDKLELDKEVTKNSEKLKNIDLSLLLRPAADIRPEAAQYCVQK   1360
SNP179068992_2.seq   FAGEPEHVINFFFMLAEEVREIMSQLGFRTINEMVGRSDKLELDKEVTKNSEKLKNIDLSLLLRPAADIRPEAAQYCVQK   1360

SNP179068992_1.seq   QDHGLDMALDNKLITLSNAALEKGLPVYIETPICNVNRAVGTMLSHEVTKRYHMAGLPADTIHIKLNGSAGQSLGAFLCP   1440
SNP179068992_2.seq   QDHGLDMALDNKLITLSNAALEKGLPVYIETPICNVNRAVGTMLSHEVTKRYHMAGLPADTIHIKLNGSAGQSLGAFLCP   1440

SNP179068992_1.seq   GITLELEGDSNDYVGKGLSGGKIVVYPPKGSNFDPKENIVIGNVALYGATVGEAYFNGMAAERFCVRNSGAKAVVEGVGD   1520
SNP179068992_2.seq   GITLELEGDSNDYVGKGLSGGKIVVYPPKGSNFDPKENIVIGNVALYGATVGEAYFNGMAAERFCVRNSGAKAVVEGVGD   1520

SNP179068992_1.seq   HGCEYMTGGTVVVLGKTGRNFAAGMSGGIAYVLDVDATFQSRCNPELVDLDKVEEEEDIMTLRVMIQQHQRHTNSQLARE   1600
SNP179068992_2.seq   HGCEYMTGGTVVVLGKTGRNFAAGMSGGIAYVLDVDATFQSRCNPELVDLDKVEEEEDIMTLRVMIQQHQRHTNSQLARE   1600

SNP179068992_1.seq   VLADFDNILPKFVKVFPRDYKRILASMKEEKIAKEAASRAANEADEQEEADLIKKDAFEELKKLAAMSSNGKDKVSETEL   1680
SNP179068992_2.seq   VLADFDNILPKFVKVFPRDYKRILASMKEEKIAKEAASRAANEADEQEEADLIKKDAFEELKKLAAMSSNGKDKVSETEL   1680

SNP179068992_1.seq   PTRPTRVPDAVKNRGFIIYEREGVSYRDPNVRMNDWKEVMEEAKPSPLLKTQSARCMDCGTPFCHQENSGCPLGNKIPEF   1760
SNP179068992_2.seq   PTRPTRVPDAVKNRGFIIYEREGVSYRDPNVRMNDWKEVMEEAKPSPLLKTQSARCMDCGTPFCHQENSGCPLGNKIPEF   1760

SNP179068992_1.seq   NELVYQNRWREALDRLLETNNFPEFTGRVCPAPCEGSCVLGIIENPVSIKNIECSIIDKAFEEGWMVPRPPLRRTGKRVA   1840
SNP179068992_2.seq   NELVYQNRWREALDRLLETNNFPEFTGRVCPAPCEGSCVLGIIENPVSIKNIECSIIDKAFEEGWMVPRPPLRRTGKRVA   1840

SNP179068992_1.seq   IVGSGPAGLAAADQLNRMGHFVTVFERADRIGGLMMYGVPNMKTDKVDIVQRRVDLMAKEGVNFVVNANVGKDPMYSLDR   1920
SNP179068992_2.seq   IVGSGPAGLAAADQLNRMGHFVTVFERADRIGGLMMYGVPNMKTDKVDIVQRRVDLMAKEGVNFVVNANVGKDPMYSLDR   1920

SNP179068992_1.seq   LREENDAIVLAVGATKPRDLPVPGRELSGVHFAMEFLHANTKSLLDSNLEDGKYISAKGKKVVVIGGGDTGTDCIGTSIR   2000
SNP179068992_2.seq   LREENDAIVLAVGATKPRDLPVPGRELSGVHFAMEFLHANTKSLLDSNLEDGKYISAKGKKVVVIGGGDTGTDCIGTSIR   2000

SNP179068992_1.seq   HGCTNIVNLELLPQPPRTRAPGNPWPQWPRIFRVDYGHQEAATKFGKDPRSYEVLTKRFVGDENGNVKALEVICVRWEKD   2080
SNP179068992_2.seq   HGCTNIVNLELLPQPPRTRAPGNPWPQWPRIFRVDYGHQEAATKFGKDPRSYEVLTKRFVGDENGNVKALEVICVRWEKD   2080

SNP179068992_1.seq   AGGKFQFKEIEGSEEMIEADLVLLAMGFLGPESTLSEKLGLEQDNRSNFKADYGRFSTNVNGVFAAGDCRRGQSLVVWAI   2160
SNP179068992_2.seq   AGGKFQFKEIEGSEEMIEADLVLLAMGFLGPESTLSEKLGLEQDNRSNFKADYGRFSTNVNGVFAAGDCRRGQSLVVWAI   2160

SNP179068992_1.seq   SEGRQAASQVDKYLMRDGQDNTTSLVERGKRQQDSSKQTVM                                          2201
SNP179068992_2.seq   SEGRQAASQVDKYLMRDGQDNTTSLVERGKRQQDSSKQTVM                                          2201
```

Figure S7. Amino acids coded by *CsGOGAT*.

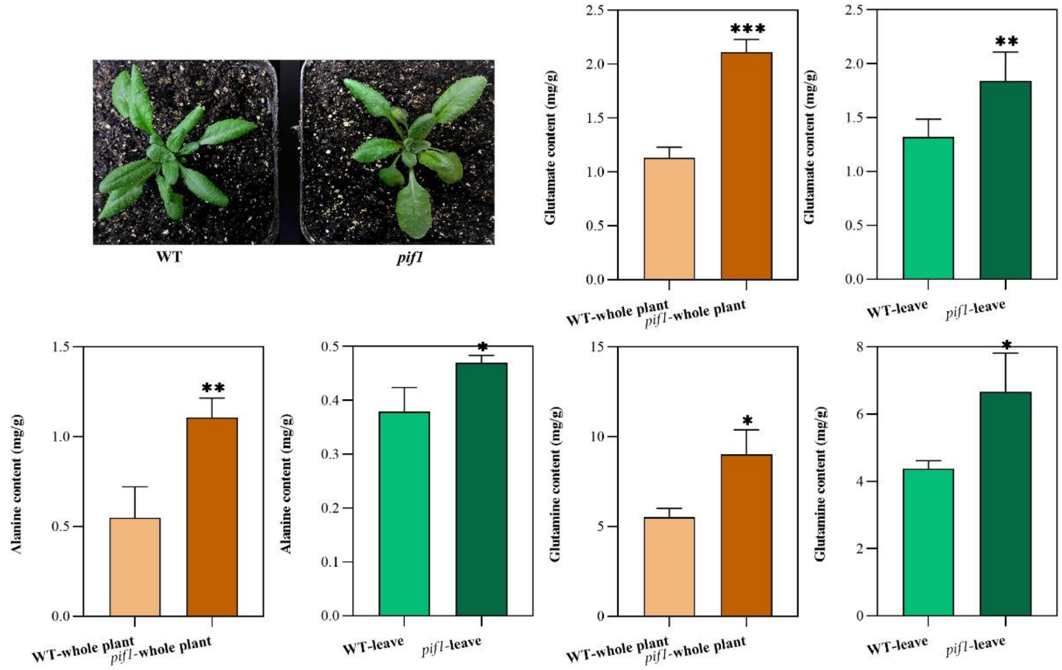

Figure S8. The phenotype of *Arabidopsis pif1* mutant and free amino acids accumulation in *pif1*. *Arabidopsis* wild type (WT) was used a negative control. Asterisks above the error bar indicate significant differences (*, $P \leq 0.05$; **, $P \leq 0.01$; ***, $P < 0.001$).
