# Supplementary material for: Comprehensive dissection of variation and accumulation of free amino acids in tea accessions
Source: Hortic Res. 2023 Dec 13;11(1):uhad263. doi: 10.1093/hr/uhad263 (PMC10833077; doi:10.1093/hr/uhad263)
Supplement: Web_Material_uhad263 [file web_material_uhad263.zip › Supplementary Table S3_ SNPs for KASP analysis.docx]

ANOVA between SNP179068992 and Theanine

| Genotype | | | | | |
| --- | --- | --- | --- | --- | --- |
|  | Sum of Squares | df | Mean Square | F | Sig. |
| Between Groups | 190.327 | 2 | 95.163 | 8.029 | 0.000493 |
| Within Groups | 1718.551 | 145 | 11.852 |  |  |
| Total | 1908.878 | 147 |  |  |  |

| Theanine | | | | | | | | |
| --- | --- | --- | --- | --- | --- | --- | --- | --- |
| (I) Genotype | | (J)Genotype | | Mean Difference (I-J) | Std. Error | Sig. | 95% Confidence Interval | |
|  |  |  |  |  |  |  | Lower Bound | Upper Bound |
|  | CC |  | TC | -1.55683 | .62317 | .014 | -2.7885 | 2.6738 |
|  |  |  | TT | -3.16624 | .84872 | .000 | -4.8437 | 3.1245 |
|  | TC |  | CC | 1.55683 | .62317 | .014 | .3252 | -.1883 |
|  |  |  | TT | -1.60941 | .89262 | .073 | -3.3736 | 1.1660 |
|  | TT |  | CC | 3.16624 | .84872 | .000 | 1.4888 | -.7671 |
|  |  |  | TC | 1.60941 | .89262 | .073 | -.1548 | .1364 |

Information of KASP analysis

| KASP | FAM | VIC | COM | Position | mutation |
| --- | --- | --- | --- | --- | --- |
| CsSNP 179068992 | GACCTCCCCTCAGAAGAACTG | CGACCTCCCCTCAGAAGAACTA | CCCTGACCACTGAATGATTCCCTAA | 179068992 | [C/T] |

|  | Genotype of 148 tea accessions |  |  |
| --- | --- | --- | --- |
| Number | Species | Origin | Genotype |
| SPG002 | *C. sinensis* (L.) O. Kuntze var*. sinensis* | Zhejiang | TC |
| SPG004 | *C. sinensis* (L.) O. Kuntze var*. sinensis* | Zhejiang | TC |
| SPG005 | *C. sinensis* (L.) O. Kuntze var*. sinensis* | Zhejiang | CC |
| SPG006 | *C. sinensis* (L.) O. Kuntze var*. sinensis* | Zhejiang | TT |
| SPG007 | *C. sinensis* (L.) O. Kuntze var*. sinensis* | Zhejiang | TC |
| SPG008 | *C. sinensis* (L.) O. Kuntze var*. sinensis* | Zhejiang | CC |
| SPG009 | *C. sinensis* (L.) O. Kuntze var*. sinensis* | Zhejiang | TC |
| SPG011 | *C. sinensis* (L.) O. Kuntze var*. sinensis* | Zhejiang | TT |
| SPG013 | *C. sinensis* (L.) O. Kuntze var*. sinensis* | Fujian | TC |
| SPG014 | *C. sinensis* (L.) O. Kuntze var*. sinensis* | Fujian | TC |
| SPG015 | *C. sinensis* (L.) O. Kuntze var*. sinensis* | Fujian | CC |
| SPG017 | *C. sinensis* (L.) O. Kuntze var*. sinensis* | Fujian | TT |
| SPG018 | *C. sinensis* (L.) O. Kuntze var*. sinensis* | Fujian | TC |
| SPG019 | *C. sinensis* (L.) O. Kuntze var*. sinensis* | Fujian | TC |
| SPG020 | *C. sinensis* (L.) O. Kuntze var*. sinensis* | Fujian | TC |
| SPG026 | *C. sinensis* (L.) O. Kuntze var*. sinensis* | Fujian | TT |
| SPG028 | *C. sinensis* (L.) O. Kuntze var*. sinensis* | Fujian | TC |
| SPG032 | *C. sinensis* (L.) O. Kuntze var*. sinensis* | Zhejiang | CC |
| SPG033 | *C. sinensis* (L.) O. Kuntze var*. sinensis* | Zhejiang | TC |
| SPG034 | *C. sinensis* (L.) O. Kuntze var*. sinensis* | Zhejiang | TT |
| SPG037 | *C. sinensis* (L.) O. Kuntze var*. sinensis* | Jiangsu | CC |
| SPG045 | *C. sinensis* (L.) O. Kuntze var*. sinensis* | Hunan | TT |
| SPG049 | *C. sinensis* (L.) O. Kuntze var*. sinensis* | Hunan | CC |
| SPG052 | *C. sinensis* (L.) O. Kuntze var*. sinensis* | Zhejiang | CC |
| SPG054 | *C. sinensis* (L.) O. Kuntze var*. sinensis* | Zhejiang | TC |
| SPG064 | *C. sinensis* (L.) O. Kuntze var*. sinensis* | Zhejiang | CC |
| SPG067 | *C. sinensis* (L.) O. Kuntze var*. sinensis* | Zhejiang | TC |
| SPG071 | *C. sinensis* (L.) O. Kuntze var*. sinensis* | Zhejiang | CC |
| SPG073 | *C. sinensis* (L.) O. Kuntze var*. sinensis* | Zhejiang | CC |
| SPG075 | *C. sinensis* (L.) O. Kuntze var*. sinensis* | Jiangxi | TC |
| SPG076 | *C. sinensis* (L.) O. Kuntze var*. sinensis* | Jiangxi | TC |
| SPG077 | *C. sinensis* (L.) O. Kuntze var*. sinensis* | Jiangxi | TC |
| SPG078 | *C. sinensis* (L.) O. Kuntze var*. sinensis* | Jiangxi | TC |
| SPG079 | *C. sinensis* (L.) O. Kuntze var*. sinensis* | Jiangxi | TT |
| SPG080 | *C. sinensis* (L.) O. Kuntze var*. sinensis* | Jiangxi | CC |
| SPG081 | *C. sinensis* (L.) O. Kuntze var*. sinensis* | Jiangxi | TC |
| SPG084 | *C. sinensis* (L.) O. Kuntze var*. sinensis* | Hunan | CC |
| SPG098 | *C. sinensis* (L.) O. Kuntze var*. sinensis* | Sichuan | TT |
| SPG99 | *C. sinensis* (L.) O. Kuntze var*. sinensis* | Anhui | CC |
| SPG101 | *C. sinensis* (L.) O. Kuntze var*. sinensis* | Anhui | CC |
| SPG105 | *C.sinensis* var. *pubilimba* Chang | Guangxi | TT |
| SPG106 | *C.sinensis* var. *pubilimba* Chang | Guangxi | CC |
| SPG107 | *C.sinensis* var. *pubilimba* Chang | Guangxi | CC |
| SPG108 | *C.sinensis* var. *pubilimba* Chang | Guangxi | CC |
| SPG110 | *C. sinensis* (L.) O. Kuntze var*. sinensis* | Guangxi | CC |
| SPG112 | *C.sinensis* var. *pubilimba* Chang | Guangxi | CC |
| SPG120 | *C. sinensis* (L.) O. Kuntze var*. sinensis* | Guangxi | CC |
| SPG126 | *C. sinensis* (L.) O. Kuntze var*. sinensis* | Guangdong | CC |
| SPG129 | *C. sinensis* var. *assamica* (Masters) Kitamura | Guangdong | TC |
| SPG130 | *C. sinensis* var. *assamica* (Masters) Kitamura | Guangdong | TC |
| SPG131 | *C. sinensis* var. *assamica* (Masters) Kitamura | Taiwan | CC |
| SPG132 | *C. sinensis* var. *assamica* (Masters) Kitamura | Hainan | CC |
| SPG134 | *C. sinensis* var. *assamica* (Masters) Kitamura | Yunnan | CC |
| SPG138 | *C. taliensis* (W. W. Smith) Melchior | Yunnan | TT |
| SPG139 | *C. sinensis* (L.) O. Kuntze var*. sinensis* | Yunnan | CC |
| SPG141 | *C. taliensis* (W. W. Smith) Melchior | Yunnan | CC |
| SPG142 | *C. sinensis* var. *assamica* (Masters) Kitamura | Yunnan | CC |
| SPG143 | *C. sinensis* (L.) O. Kuntze var*. sinensis* | Yunnan | TT |
| SPG144 | *C. sinensis* (L.) O. Kuntze var*. sinensis* | Yunnan | CC |
| SPG149 | *C. sinensis* var. *assamica* (Masters) Kitamura | Yunnan | CC |
| SPG150 | *C. sinensis* (L.) O. Kuntze var*. sinensis* | Chongqing | CC |
| SPG151 | *C. tachangensis* F. C. Zhang | Guizhou | CC |
| SPG152 | *C. tachangensis* F. C. Zhang | Guizhou | TC |
| SPG153 | *C. tachangensis* F. C. Zhang | Guizhou | TC |
| SPG157 | *C. sinensis* (L.) O. Kuntze var*. sinensis* | Yunnan | TC |
| SPG158 | *C. sinensis* var. *assamica* (Masters) Kitamura | Yunnan | TC |
| SPG161 | *C. crassicolumna* Chang | Yunnan | CC |
| SPG162 | *C. sinensis* var. *assamica* (Masters) Kitamura | Yunnan | TC |
| SPG164 | *C. muricatula* Chang | Yunnan | CC |
| SPG165 | *C. sinensis* var. *assamica* (Masters) Kitamura | Yunnan | TC |
| SPG166 | *C. taliensis* (W. W. Smith) Melchior | Yunnan | CC |
| SPG167 | *C. sinensis* var. *assamica* (Masters) Kitamura | Yunnan | CC |
| SPG168 | *C. sinensis* var. *assamica* (Masters) Kitamura | Yunnan | CC |
| SPG169 | *C. taliensis* (W. W. Smith) Melchior | Yunnan | CC |
| SPG170 | *C. crassicolumna* Chang | Yunnan | CC |
| SPG171 | *C. sinensis* var. *assamica* (Masters) Kitamura | Yunnan | CC |
| SPG174 | *C. taliensis* (W. W. Smith) Melchior | Yunnan | TC |
| SPG175 | *C. taliensis* (W. W. Smith) Melchior | Yunnan | TT |
| SPG176 | *C. sinensis* (L.) O. Kuntze var*. sinensis* | Yunnan | CC |
| SPG179 | *C. sinensis* (L.) O. Kuntze var*. sinensis* | Yunnan | CC |
| SPG184 | *C. tachangensis* F. C. Zhang | Yunnan | CC |
| SPG185 | *C. taliensis* (W. W. Smith) Melchior | Yunnan | CC |
| SPG192 | *C. sinensis* var. *assamica* (Masters) Kitamura | Yunnan | TC |
| SPG193 | *C. taliensis* (W. W. Smith) Melchior | Yunnan | TC |
| SPG198 | *C. sinensis* var. *assamica* (Masters) Kitamura | Yunnan | TC |
| SPG201 | *C. sinensis* var. *assamica* (Masters) Kitamura | Yunnan | CC |
| SPG203 | *C. sinensis* (L.) O. Kuntze var*. sinensis* | Yunnan | CC |
| SPG204 | *C.* sp | Yunnan | CC |
| SPG205 | *C. sinensis* (L.) O. Kuntze var*. sinensis* | Guangxi | TT |
| SPG206 | *C.* sp | Guangdong | TT |
| SPG207 | *C.* sp | Guangdong | CC |
| SPG209 | *C. sinensis* (L.) O. Kuntze var*. sinensis* | Sichuan | CC |
| SPG210 | *C.sinensis* var. *pubilimba* Chang | Guangxi | TT |
| SPG214 | *C.sinensis* var. *pubilimba* Chang | Guangxi | CC |
| SPG218 | *C. sinensis* var. *assamica* (Masters) Kitamura | Hainan | CC |
| SPG220 | *C. sinensis* var. *assamica* (Masters) Kitamura | Hainan | CC |
| SPG222 | *C. sinensis* var. *assamica* (Masters) Kitamura | Hainan | CC |
| SPG224 | *C. sinensis* (L.) O. Kuntze var*. sinensis* | Chongqing | TT |
| SPG227 | *C. sinensis* var. *assamica* (Masters) Kitamura | Yunnan | TT |
| SPG228 | *C. sinensis* var. *assamica* (Masters) Kitamura | Taiwan | TT |
| SPG229 | *C. sinensis* var. *assamica* (Masters) Kitamura | Hainan | CC |
| SPG231 | *C. sinensis* var. *assamica* (Masters) Kitamura | Hainan | CC |
| SPG232 | *C. sinensis* var. *assamica* (Masters) Kitamura | Hainan | TT |
| SPG233 | *C. sinensis* var. *assamica* (Masters) Kitamura | Hainan | CC |
| SPG237 | *C. sinensis* (L.) O. Kuntze var*. sinensis* | Zhejiang | CC |
| SPG241 | *C. sinensis* (L.) O. Kuntze var*. sinensis* | Fujian | TC |
| SPG242 | *C. sinensis* (L.) O. Kuntze var*. sinensis* | Fujian | TC |
| SPG246 | *C. sinensis* (L.) O. Kuntze var*. sinensis* | Hunan | CC |
| SPG258 | *C. sinensis* (L.) O. Kuntze var*. sinensis* | Zhejiang | TT |
| SPG260 | *C. sinensis* (L.) O. Kuntze var*. sinensis* | Zhejiang | CC |
| SPG266 | *C. sinensis* (L.) O. Kuntze var*. sinensis* | Anhui | CC |
| SPG267 | *C. sinensis* (L.) O. Kuntze var*. sinensis* | Anhui | TC |
| SPG270 | *C. sinensis* (L.) O. Kuntze var*. sinensis* | Fujian | CC |
| SPG271 | *C. sinensis* (L.) O. Kuntze var*. sinensis* | Fujian | CC |
| SPG277 | *C. sinensis* (L.) O. Kuntze var*. sinensis* | Fujian | TC |
| SPG282 | *C. sinensis* (L.) O. Kuntze var*. sinensis* | Fujian | CC |
| SPG283 | *C. sinensis* (L.) O. Kuntze var*. sinensis* | Fujian | CC |
| SPG284 | *C. sinensis* (L.) O. Kuntze var*. sinensis* | Fujian | TC |
| SPG293 | *C. sinensis* (L.) O. Kuntze var*. sinensis* | Fujian | TC |
| SPG294 | *C. sinensis* (L.) O. Kuntze var*. sinensis* | Guangdong | TC |
| SPG295 | *C.sinensis* var. *pubilimba* Chang | Guangdong | TC |
| SPG297 | *C. sinensis* (L.) O. Kuntze var*. sinensis* | Sichuan | TC |
| SPG303 | *C.sinensis* var. *pubilimba* Chang | Guangxi | TC |
| SPG307 | *C. sinensis* (L.) O. Kuntze var*. sinensis* | Zhejiang | CC |
| SPG308 | *C. sinensis* var. *assamica* (Masters) Kitamura | Zhejiang | TC |
| SPG318 | *C. sinensis* (L.) O. Kuntze var*. sinensis* | Zhejiang | TC |
| SPG325 | *C. sinensis* (L.) O. Kuntze var*. sinensis* | Anhui | TC |
| SPG372 | *C. ptilophylla* Chang | Guangdong | CC |
| SPG373 | *C. ptilophylla* Chang | Guangdong | CC |
| SPG380 | *C. sinensis* (L.) O. Kuntze var*. sinensis* | Taiwan | TT |
| SPG382 | *C. sinensis* (L.) O. Kuntze var*. sinensis* | Guangdong | CC |
| SPG383 | *C. sinensis* (L.) O. Kuntze var*. sinensis* | Guangdong | CC |
| SPG384 | *C. sinensis* (L.) O. Kuntze var*. sinensis* | Zhejiang | TC |
| SPG385 | *C.sinensis* var. *pubilimba* Chang | Guizhou | TC |
| SPG387 | *C. sinensis* (L.) O. Kuntze var*. sinensis* | Sichuan | TC |
| SPG388 | *C. sinensis* (L.) O. Kuntze var*. sinensis* | Sichuan | CC |
| SPG389 | *C. sinensis* (L.) O. Kuntze var*. sinensis* | Sichuan | CC |
| SPG390 | *C. sinensis* (L.) O. Kuntze var*. sinensis* | Sichuan | TC |
| SPG392 | *C. sinensis* (L.) O. Kuntze var*. sinensis* | Chongqing | TC |
| SPG394 | *C. sinensis* (L.) O. Kuntze var*. sinensis* | Chongqing | CC |
| SPG396 | *C. sinensis* (L.) O. Kuntze var*. sinensis* | Sichuan | CC |
| SPG397 | *C. sinensis* (L.) O. Kuntze var*. sinensis* | Sichuan | TC |
| SPG398 | *C. sinensis* (L.) O. Kuntze var*. sinensis* | Chognqing | CC |
| SPG399 | *C. sinensis* (L.) O. Kuntze var*. sinensis* | Sichuan | CC |
| SPG400 | *C. sinensis* (L.) O. Kuntze var*. sinensis* | Hubei | TC |
| SPG401 | *C. sinensis* (L.) O. Kuntze var*. sinensis* | Hubei | CC |
| SPG402 | *C. sinensis* (L.) O. Kuntze var*. sinensis* | Shanxi | CC |
| BJG | *C. sinensis* (L.) O. Kuntze var*. sinensis* | Fujian | TC |
| LJ43 | *C. sinensis* (L.) O. Kuntze var*. sinensis* | Zhejiang | TC |
| G1 | *C. sinensis* (L.) O. Kuntze var*. sinensis* |  | CC |
| G2 | *C. sinensis* (L.) O. Kuntze var*. sinensis* |  | CC |
| Y1 | *C. sinensis* (L.) O. Kuntze var*. sinensis* |  | TC |
| Y2 | *C. sinensis* (L.) O. Kuntze var*. sinensis* |  | TC |
