# Supplementary material for: Comprehensive dissection of variation and accumulation of free amino acids in tea accessions
Source: Hortic Res. 2023 Dec 13;11(1):uhad263. doi: 10.1093/hr/uhad263 (PMC10833077; doi:10.1093/hr/uhad263)
Supplement: Web_Material_uhad263 [file web_material_uhad263.zip › Supplementary Table S5_ The sequences of primers for qRT-PCR.docx]

The sequences of primers for qRT-PCR

| RT-PCR primers | | |
| --- | --- | --- |
| Gene name | F Forward primers (5’-3’) | Reverse primers (5’-3’) |
| GAPDH | GGCATCGTTGAGGGTCT | CAGTGGGAACACGGAAAGC |
| *CsPIF1* | TCTACCGTGACTTCTACGCTGA | GATTCCCTCACCGCCTTGTTCG |
